# Efficacy and Safety of DL-3-n-Butylphthalide in the Treatment of Poststroke Cognitive Impairment: A Systematic Review and Meta-Analysis

**DOI:** 10.3389/fphar.2021.810297

**Published:** 2022-01-25

**Authors:** Xueming Fan, Wei Shen, Liuding Wang, Yunling Zhang

**Affiliations:** Xiyuan Hospital, China Academy of Chinese Medical Sciences, Beijing, China

**Keywords:** Dl-3-n-butylphthalide, poststroke cognitive impairment, systematic review, cognitive function, potential mechanism

## Abstract

**Background:** Poststroke cognitive impairment (PSCI) is a common complication observed after stroke. Current pharmacologic therapies have no definitive evidence for cognitive recovery or disease progression. Recent studies have verified the positive effect of DL-3-n-butylphthalide (NBP). However, the clinical efficacy and safety are still unclear. The aim of this study was to assess the efficacy of NBP and its harmful effect in the treatment of PSCI.

**Method:** Eligible randomized controlled trials (RCTs) were retrieved from inception to June 2021 from seven medical databases and two clinical registries. The revised Cochrane risk of bias tool (RoB 2.0) was used for methodological quality. RevMan v5.4.1 from Cochrane Collaboration was used for statistical analysis, and Hartung-Knapp-Sidik-Jonkman (HKSJ) method was used for post hoc testing depend on the number of studies. This study has been submitted to PROSPERO with registration number is CRD42021274123.

**Result:** We identified 26 studies with a total sample size of 2,571 patients. The results of this study showed that NBP as monotherapy or combination therapy had better performance in increasing the MoCA (monotherapy: SMD_N_ = 1.05, 95% CI [0.69, 1.42], *p* < 0.00001; SMD_P_ = 1.06, 95% CI [0.59, 1.52], *p* < 0.00001. combination: SMD_O_ = 0.81, 95% CI [0.62, 1.01], *p* < 0.00001; SMD_N_ = 0.90, 95% CI [0.46, 1.33], *p* < 0.0001; SMD_D_ = 1.04, 95% CI [0.71, 1.38], *p* < 0.00001), MMSE (monotherapy: MD_N_ = 4.89, 95% CI [4.14, 5.63]), *p* < 0.00001). combination: SMD_O_ = 1.26, 95% CI [0.97, 1.56], *p* < 0.00001; SMD_C_ = 1.63, 95% CI [1.28, 1.98], *p* < 0.00001; SMD_N_ = 2.13, 95% CI [1.52, 2.75], *p* < 0.00001) and BI (monotherapy: MD_N_ = 13.53, HKSJ 95% CI [9.84, 17.22], *p* = 0.014. combination: SMD_O_ = 2.24, HKSJ 95%CI [0.37, 4.11], *p* = 0.032; SMD_C_ = 3.36, 95%CI [2.80, 3.93], *p* < 0.00001; SMD_D_ = 1.48, 95%CI [1.13, 1.83], *p* < 0.00001); and decreasing the NIHSS (monotherapy: MD_N_ = −3.86, 95% CI [−5.22, −2.50], *p* < 0.00001. combination: SMD_O_ = −1.15, 95% CI [−1.31, −0.98], *p* < 0.00001; SMD_C_ = −1.82, 95% CI [−2.25, −1.40], *p* < 0.00001) and CSS (combination: MD_O_ = −7.11, 95% CI [−8.42, −5.80], *p* < 0.00001), with no serious adverse reactions observed. The funnel plot verified the possibility of publication bias.

**Conclusion:** NBP maintains a stable pattern in promoting the recovery of cognitive function and abilities of daily living, as well as reducing the symptoms of neurological deficits. However, there is still a need for more high-quality RCTs to verify its efficacy and safety.

## 1 Introduction

Poststroke cognitive impairment (PSCI) is a common complication after stroke (Zhang and Bi, 2020). Approximately 9–30% of patients develop dementia within 1 year (Kim et al., 2020), and 35.6% of patients develop cognitive impairment within 5 years after an event (Rohde et al., 2019). The impairment of cognitive function after stroke involves multiple cognitive fields, with attention and executive dysfunction as the core symptoms (Carlson et al., 2009; Aam et al., 2020), which not only affects the quality of daily life and the ability to live independently (Shi et al., 2018; Ding et al., 2019) but also brings a heavy burden to medical institutions and nursing staff (Black et al., 2018). Brain plasticity refers to the ability of the brain to spontaneously repair nerves based on structural and functional changes after stroke (Mcewen, 2016; Ossenkoppele et al., 2020; Regenhardt et al., 2020). Drug therapy can promote the recovery of neurological function and reduce cognitive impairment. Although the consensus of experts recommends the use of cholinesterase inhibitors, memantine, nimodipine and oxiracetam for the treatment of PSCI (Sun et al., 2014; Dong et al., 2017; Wang et al., 2021), there is insufficient evidence to support drug therapy in this process (Quinn et al., 2021). Moreover, current evidence is based on clinical studies of Alzheimer’s disease (AD) and vascular cognitive impairment (VCI), and there is no convincing clinical evidence that current pharmacologic therapies can promote cognitive recovery or prevent disease progression in the treatment of PSCI (Brainin et al., 2015).

DL-3-n-butylphthalide (NBP) is a new drug developed independently in China that acts on multiple pathological links of acute ischemic stroke (Wang et al., 2018). In addition to promoting the recovery of cognitive function after stroke, recent clinical studies have demonstrated that NBP can improve the oxidative stress response of the nervous system (Lv et al., 2018), inhibit neuronal apoptosis and autophagy (Xu J et al., 2017), regulate central cholinergic function (Zhao et al., 2014; Sun et al., 2020) and promote neuroplasticity (Yang et al., 2015). A randomized controlled study involving 281 patients with subcortical nondementia VCI also showed that 6 months of NBP treatment effectively improved the cognitive and overall function of patients (Jia et al., 2016). The expert Consensus on the Management of Cognitive Impairment after Stroke (2021) recommends the use of NBP as a drug therapy for PSCI (Class Ⅱ, Level B evidence) (Wang et al., 2021), but current clinical studies are mostly small sample randomized controlled trials, and the clinical efficacy and safety of NBP are still uncertain. In 2018, Feng Zhiguo et al. (Feng et al., 2018) systematically evaluated the clinical effectiveness of NBP, and the results showed that NBP can significantly improve cognitive function and activities of daily living of patients after stroke. However, because of the simplicity of the search strategy, the possibility of incomplete search and publication bias, and the publication of new clinical studies, it is necessary to update the systematic review of the clinical efficacy of NBP in the treatment of PSCI to provide new evidence references for the clinical treatment of this disease.

## 2 Materials and Methods

### 2.1 Protocol Registration

This systematic review has been registered on the PROSPERO International prospective register of systematic reviews (ID = CRD42021274123).

### 2.2 Search Methods

Seven major medical databases were comprehensively retrieved, including the China National Knowledge Infrastructure (CNKI), Wanfang Database, China Science and Technology Journal Database (VIP) and Chinese Biomedical literature Service System (SinoMed) in Chinese and PubMed, EMBASE, and Cochrane Library in English. Ongoing or unpublished studies registered on clinical registration websites, such as the Chinese Clinical Trial Registry and Clinical Trials.gov, were also searched to collect overall information. The retrieval time was limited from database establishment to June 2021, with the combination of medical subject heading and free-text terms.

### 2.3 Inclusion Criteria

#### 2.3.1 Types of Studies

Eligible randomized controlled trials (RCTs) evaluating the clinical efficacy and safety of NBP in the treatment of PSCI were included. All studies were full-text literature in English and Chinese, regardless of study site, publication date or research status.

#### 2.3.2 Types of Participants

We included studies in patients definitively diagnosed with PSCI (Wang et al., 2021). In addition, RCTs that met the first diagnosis of stroke and the second diagnosis of cognitive impairment, emphasizing that cognitive impairment occurred after stroke, were also included.

#### 2.3.3 Types of Interventions

The intervention group was treated with oral NBP alone or in combination with other positive control drugs (drug dosage, dosage form and course of treatment were consistent with the control group). The control group contained the same positive control drugs recommended by the expert consensus (Wang et al., 2021), such as cholinesterase inhibitors, memantine, nimodipine and oxiracetam. Both groups were treated continuously with no limitation of treatment course.

#### 2.3.4 Types of Outcomes

The primary outcome was cognitive function evaluation, and the eligible RCTs contained at least one of the following cognitive scales: Mini-Mental State Examination (MMSE), Montreal Cognitive Assessment (MoCA), Alzheimer’s Disease Assessment Scale-Cognitive Subscale (ADAS-Cog), Vascular Dementia Assessment Scale-Cognitive Subscale (VADAS-Cog), Wechsler Memory Scale (WMS), and Hastgawa Dementia Scale (HDS).

The secondary outcome included abilities of daily living, evaluated by Barthel index (BI) and Activities of Daily Living scale (ADL); neurological impairment, assessed by National Institutes of Health Stroke Scale (NIHSS) and China Stroke Scale (CSS).

Safety indicators, such as the occurrence of adverse events or adverse reactions, were used to analyze the clinical safety of NBP.

### 2.4 Exclusion Criteria

We excluded studies with incorrect or incomplete data. For duplicated studies, we included only the most recent and comprehensive information.

### 2.5 Study Selection and Data Extraction

Endnote X9 was used for literature management. One reviewer (Xueming Fan) downloaded the literature information identified from the search strategy and eliminated duplicates according to the titles and authors. Then, two researchers (Xueming Fan and Liuding Wang) independently screened and extracted data information according to the eligibility criteria, and a third researcher (Wei Shen) adjudicated any disagreement. Through reading the titles, abstracts and full text of the literature, the final number of studies that might be included was determined, and the reasons for exclusion were recorded. According to the predesigned standardized information extraction table, the relevant data of the qualified literature were extracted, including the first author, study title, publication date, sample size, gender, age, intervention measures, drug dose, comorbid conditions, course of treatment, follow-ups, outcome measures, adverse reactions.

### 2.6 Risk of Bias Assessment of Included Studies

The methodological quality of the included studies was evaluated by two researchers (Xueming Fan and Liuding Wang) using the revised Cochrane risk of bias tool (RoB 2.0) (Sterne et al., 2019). Specific evaluation contents included randomization process, deviations from intended interventions, missing outcome data, measurement of the outcome, and selection of the reported result. Through reading the full texts of the studies, the risk of bias for each domain was judged as high risk, low risk and some concerns. If all domains showed low risk, the overall risk of bias was considered low; If any of the above domains showed some concerns with no “high risk” field, the overall risk of bias was determined as some concerns; If high risk was reached for at least one domain, or judgement in multiple domains included some concerns, the overall risk of bias was considered high. A third researcher (Wei Shen) convened discussions or meetings to set rules when a disagreement arose between two reviewers.

### 2.7 Data Synthesis and Statistical Analysis

RevMan v5.4.1 provided by the Cochrane Collaboration was used for statistical analysis of the extracted clinical information. Relative risk (RR) analysis statistics for dichotomous outcomes and mean difference (MD) or standard mean difference (SMD) analysis statistics for continuous outcomes were applied according to different data types, both of which were calculated by 95% confidence intervals (CIs). Cochrane’s *X* ^2^ and *I* ^2^ tests were used to determine heterogeneity. Considering that the intervention measures of the control group had a certain influence on the results of the study, we conducted subgroup analysis according to different measures, and carried out SMD analysis statistics in these studies with continuous outcomes. Results were pooled when there were two or more trials with a common outcome. In view of the model selection based on heterogeneity results should be avoided, for studies that could not obtain complete information, we selected random effects model for analysis. Hartung-Knapp-Sidik-Jonkman (HKSJ) method was used for post hoc testing with a predesigned program (Inthout et al., 2014), for HKSJ has more sufficient error rate compared with DerSimonian and Laird’s (DL) applied in RevMan software, especially when the number of studies is small (Inthout et al., 2014). In addition, if the number of studies was ≥ 10, funnel plot analysis was used to detect publication bias.

### 2.8 Certainty Assessment of Evidence

Two reviewers (Xueming Fan and Liuding Wang) evaluated the certainty of evidence using the Grading of Recommendations, Assessment, Development, and Evaluations (GRADE) system (Guyatt et al., 2008), which identified the quality of outcome evidence as high, moderate, low, or very low. The certainty can be downgraded for five reasons (risk of bias, imprecision, inconsistency, indirectness, and publication bias) and upgraded for three reasons (large magnitude of an effect, dose-response gradient, and effect of plausible residual confounding). Besides, any disagreements were resolved through a third reviewer (Wei Shen).

## 3 Result

### 3.1 Study Selection

**FIGURE 1:**
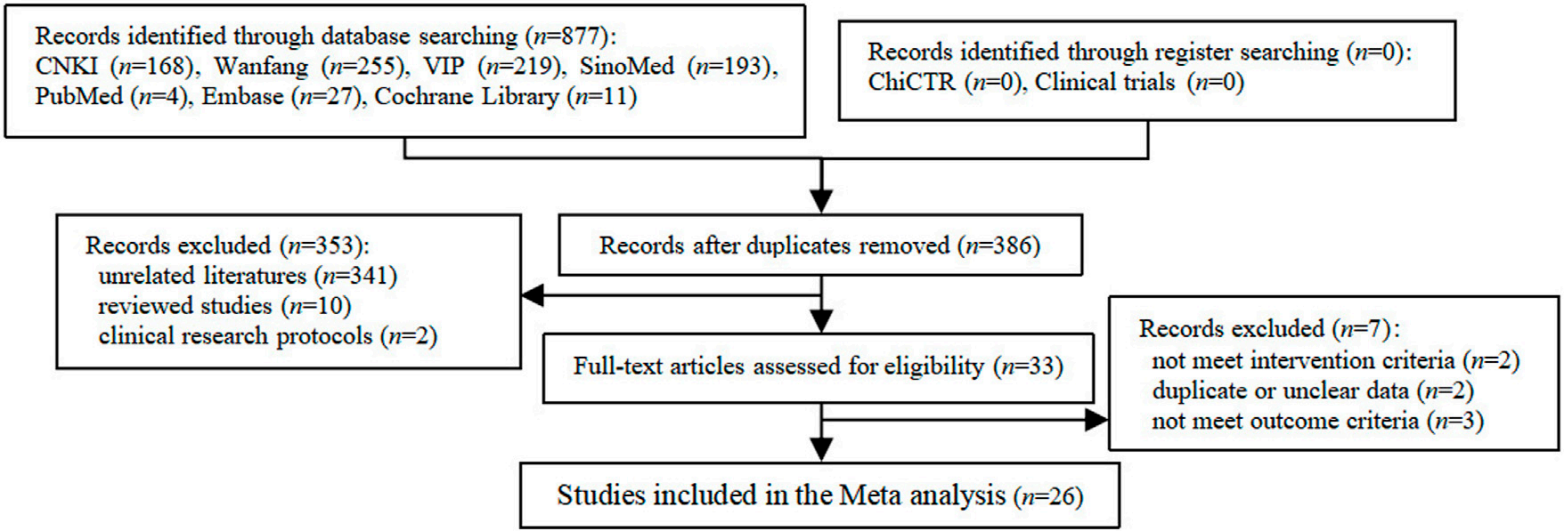
Flow chart of study inclusion.

A total of 877 studies were retrieved in our search strategy (Supplementary Table S1), and 491 duplicate studies were excluded. After screening the retrieved titles and abstracts, 341 unrelated studies, 10 reviewed studies and 2 clinical research protocols were removed. Thirty-three full-text studies remained and were evaluated for eligibility. Seven studies were excluded because they did not meet the intervention or outcome criteria, including duplicate or unclear data (Supplementary Table S2). Finally, 26 relevant studies (Yang, 2011; Xia, 2013; Fu, 2014; You, 2014; Su et al., 2015; Xing and Abudushalamu, 2015; Zhao, 2015; Cheng et al., 2016; Ji et al., 2016; Qi et al., 2016; Zhu et al., 2016; Li, 2017; Wang, 2017; Xu H et al., 2017; Li et al., 2018; Liu et al., 2018; Sun, 2018; Wang, 2018; Yang, 2018; Bai, 2019; Li X et al., 2019; Liu, 2019; Liu et al., 2019; Luo, 2019; Chen et al., 2020; Fang, 2021) were included in our study. The specific literature screening process is shown in Figure 1.

### 3.2 Study Inclusion Characteristics

We included 26 randomized controlled trials, with a total sample size of 2,571 patients. The mean age of participants in each study ranged from 50 to 80 years. All the studies were conducted in Chinese and published between 2011 and 2021. The comorbid conditions of participants were mostly hypertension, diabetes mellitus, hyperlipoidemia and coronary heart disease. The course of treatment ranged from 0.5 to 6 months. Ten studies reported comparisons of NBP with other positive control drugs, and 16 studies reported combination therapy with NBP. Cognitive function was assessed by Montreal Cognitive Assessment (MoCA) in 18 studies and Mini-Mental State Examination (MMSE) in 16 studies. The basic characteristics of the 26 included studies are summarized in detail in Table 1.

**TABLE 1:** Characteristics of included studies.

Study ID	Sample size		Mean age (year)		Male/Female (male%)		Intervention		Drug dose	Comorbid conditions (count)		Course of treatment (month)	Outcomes
	Trial	Control	Trial	Control	Trial	Control	Trial	Control		Trial	Control		
Fang Fang 2021 Fang. (2021)	60	60	69.20 ± 4.50	69.10 ± 4.60	35/25 (58%)	36/24 (60%)	NBP + Citicoline	Citicoline	NBP: 200 mg, Tid, Citicoline: 100 mg, Tid	NR		0.5	(b) (c) (e)
Chen Shouqiang 2020 Chen et al. (2020)	79	79	65.13 ± 7.24	64.59 ± 6.38	40/39 (51%)	41/38 (52%)	NBP + Donepezil	Donepezil	NBP: 200 mg, Tid, Donepezil: 5 mg, Qd	HTN: 24; CHD: 10; HL: 15; DM: 9	HTN: 25; CHD: 11; HL: 15; DM: 9	6.0	(a) (c)
Bai Xingyong 2019 Bai. (2019)	39	39	61.21 ± 9.87	61.98 ± 8.94	21/18 (54%)	20/19 (51%)	NBP	Nimodipine	NBP: 200 mg, Tid, Nimodipine: 30-120 mg/d	NR		1.0	(a) (c) (e) (g)
Li Xinian 2019 Li X et al. (2019)	32	32	62.20 ± 11.40	61.40 ± 10.10	18/14 (56%)	19/13 (59%)	NBP + Oxiracetam	Oxiracetam	NBP: 200 mg, Tid, Oxiracetam: 800 mg, Tid	NR		0.5	(a) (b) (c) (g)
Luo Bang 2019 Luo. (2019)	61	56	63.72 ± 8.93	64.17 ± 9.30	35/26 (57%)	32/24 (57%)	NBP + Oxiracetam	Oxiracetam	NBP: 200 mg, Tid, Oxiracetam: 800 mg, Tid	HTN: 21; DM: 16; CHD: 9	HTN: 22; DM: 13; CHD: 10	1.0	(a) (c) (g)
Liu Shu 2019 Liu et al. (2019)	49	48	62.00 ± 7.40	62.60 ± 7.10	27/22 (55%)	28/20 (58%)	NBP + Oxiracetam	Oxiracetam	NBP: 200 mg, Tid, Oxiracetam: 800 mg, Tid	NR		1.0	(b) (c) (e) (g)
Liu Qie 2019 Liu. (2019)	40	40	NR	NR	NR	NR	NBP	Nimodipine	NBP: 200 mg, Qid, Nimodipine: 30 mg, Tid	NR		2.0	(a) (b)
Liu Cuiping 2018 Liu et al. (2018)	51	47	66.70 ± 7.40	67.20 ± 7.70	26/25 (51%)	24/23 (51%)	NBP	Nimodipine	NBP: 200 mg, Qid, Nimodipine: 60 mg, Tid	NR		3.0	(a)
Sun Longyin 2018 Sun. (2018)	120	120	62.50 ± 8.54	63.40 ± 9.17	72/48 (60%)	66/54 (55%)	NBP + Oxiracetam	Oxiracetam	NBP: 200 mg, Tid, Oxiracetam: 800 mg, Tid	NR		3.0	(a) (e)
Wang Yuetao 2018 (Wang. 2018)	45	45	63.70 ± 6.10		52/38 (58%)		NBP + Oxiracetam	Oxiracetam	NBP: 200 mg, Tid, Oxiracetam: 800 mg, Tid	NR		3.0	(a) (b) (c) (g)
Yang Yisen 2018 Yang. (2018)	45	45	63.70 ± 6.30		54/36 (60%)		NBP + Oxiracetam	Oxiracetam	NBP: 200 mg, Tid, Oxiracetam: 800 mg, Tid	HTN: 31; DM: 19; CHD: 17		3.0	(a) (b) (c) (g)
Li Wei 2018 Li et al. (2018)	54	54	60.62 ± 19.35	59.94 ± 20.13	29/25 (54%)	31/23 (57%)	NBP + Oxiracetam	Oxiracetam	NBP: 200 mg, Tid, Oxiracetam: 800 mg, Tid	HTN: 22; CHD: 12; HL: 14; DM: 14	HTN: 23; CHD: 11; HL: 15; DM: 12	1.0	(a) (f) (g)
Li Peiyu 2017 Li. (2017)	50	50	72.50 ± 6.90	71.70 ± 6.50	27/23 (54%)	29/21 (58%)	NBP + Oxiracetam	Oxiracetam	NBP: 200 mg, Tid, Oxiracetam: 800 mg, Tid	NR		3.0	(a) (b) (c)
Wang Kuanhong 2017 Wang. (2017)	33	32	65.90 ± 4.50	65.20 ± 4.10	19/14 (58%)	18/14 (56%)	NBP + Nimodipine	Nimodipine	NBP: 200 mg, Tid, Nimodipine: 30 mg, Tid	HTN: 14; DM: 11; CHD: 8	HTN: 13; DM: 12; CHD: 7	2.0	(b) (c)
Xu Hongqiang 2017 Xu H et al. (2017)	104	104	63.14 ± 9.59	63.68 ± 10.18	59/45 (57%)	62/42 (60%)	NBP + Oxiracetam	Oxiracetam	NBP: 200 mg, Tid, Oxiracetam: 800 mg, Tid	NR		3.0	(a) (e) (g)
Zhu Jie 2016 Zhu et al. (2016)	26	22	60.38 ± 2.54	61.56 ± 2.34	20/6 (77%)	14/8 (64%)	NBP + Citicoline	Citicoline	NBP: 200 mg, Tid, Citicoline: 200 mg, Tid	NR		6.0	(a) (b) (d) (g)
Cheng Wenyao 2016 Cheng et al. (2016)	48	48	65.20 ± 8.90	64.60 ± 9.20	31/17 (65%)	32/16 (67%)	NBP	Nimodipine	NBP: 200 mg, Tid, Nimodipine: 40 mg, Tid	NR		2.0	(b) (c)
Ji Zhi 2016 Ji et al. (2016)	52	52	64.80 ± 10.80	64.00 ± 10.90	28/24 (54%)	32/20 (62%)	NBP + Oxiracetam	Oxiracetam	NBP: 200 mg, Tid, Oxiracetam: 800 mg, Tid	HTN: 31; DM: 19; CHD: 10	HTN: 33; DM: 20; CHD: 8	3.0	(a) (e) (g)
Qi Weinan 2016 Qi et al. (2016)	44	44	67.33 ± 7.22	66.97 ± 7.35	24/20 (55%)	22/22 (50%)	NBP + Nimodipine	Nimodipine	NBP: 200 mg, Tid, Nimodipine: 30 mg, Tid	NR		3.0	(b) (c) (g)
Su Xudong 2015 Su et al. (2015)	31	31	63.00 ± 5.20	62.00 ± 7.90	19/12 (61%)	21/10 (68%)	NBP	Nimodipine	NBP: 200 mg, Tid, Nimodipine: 40 mg, Tid	NR		6.0	(a) (b) (c) (g)
Xing Geli 2015 Xing and Abudushalamu. (2015)	49	49	56.50 ± 5.30	58.70 ± 5.10	26/23 (53%)	28/21 (57%)	NBP	Nimodipine	NBP: 400 mg, Tid, Nimodipine: 50 mg, Tid	HTN: 33; DM: 19; CHD: 14	HTN: 37; DM: 18; CHD: 16	6.0	(b) (c) (g)
Zhao Haiyan 2015 Zhao. (2015)	41	40	58.14 ± 7.09	59.33 ± 7.00	24/17 (59%)	22/18 (55%)	NBP	Piracetam	NBP: 200 mg, Tid, Piracetam: 1,200 mg, Tid	NR		1.0	(a) (g)
Fu Yong 2014 Fu, (2014)	35	35	56.44 ± 2.17		35/35 (50%)		NBP	Nimodipine	NBP: 200 mg, Tid, Nimodipine: 30 mg, Tid	NR		3.0	(b)
You Guoqin 2014 You. (2014)	45	44	56.80 ± 8.50		43/46 (48%)		NBP + Nimodipine	Nimodipine	NBP: 200 mg, Tid, Nimodipine: 30 mg, Tid	NR		3.0	(a) (c) (g)
Xia Deyu 2013 Xia. (2013)	30	30	52–75	53–74	16/14 (53%)	17/13 (57%)	NBP	Nimodipine	NBP: 200 mg, Tid, Nimodipine: 40 mg, Tid	NR		3.0	(b) (g)
Yang Xiaoli 2011 (Yang. 2011)^a^	31	31	62.60 ± 7.70	60.40 ± 7.00	23/20 (70%)	22/11 (67%)	NBP	Nimodipine	NBP: 200 mg, Tid, Nimodipine: 40 mg, Tid	NR		6.0	(a) (b) (d) (g)

Abbreviations: NR, Not Reported; HTN, Hypertension; DM, Diabetes Mellitus; HL, Hyperlipoidemia; CHD, Coronary Heart Disease; (a) Montreal Cognitive Assessment (MoCA); (b) Mini-Mental State Examination (MMSE); (c) Barthel Index (BI); (d) Abilities of daily living scale (ADL); (e) National Institutes of Health Stroke Scale (NIHSS); (f) China Stroke Scale (CSS); (g) Adverse events (AE).

a The data of age and gender after shedding were missing and included the total data for analysis.

### 3.3 Risk of Bias Assessment

**FIGURE 2:**
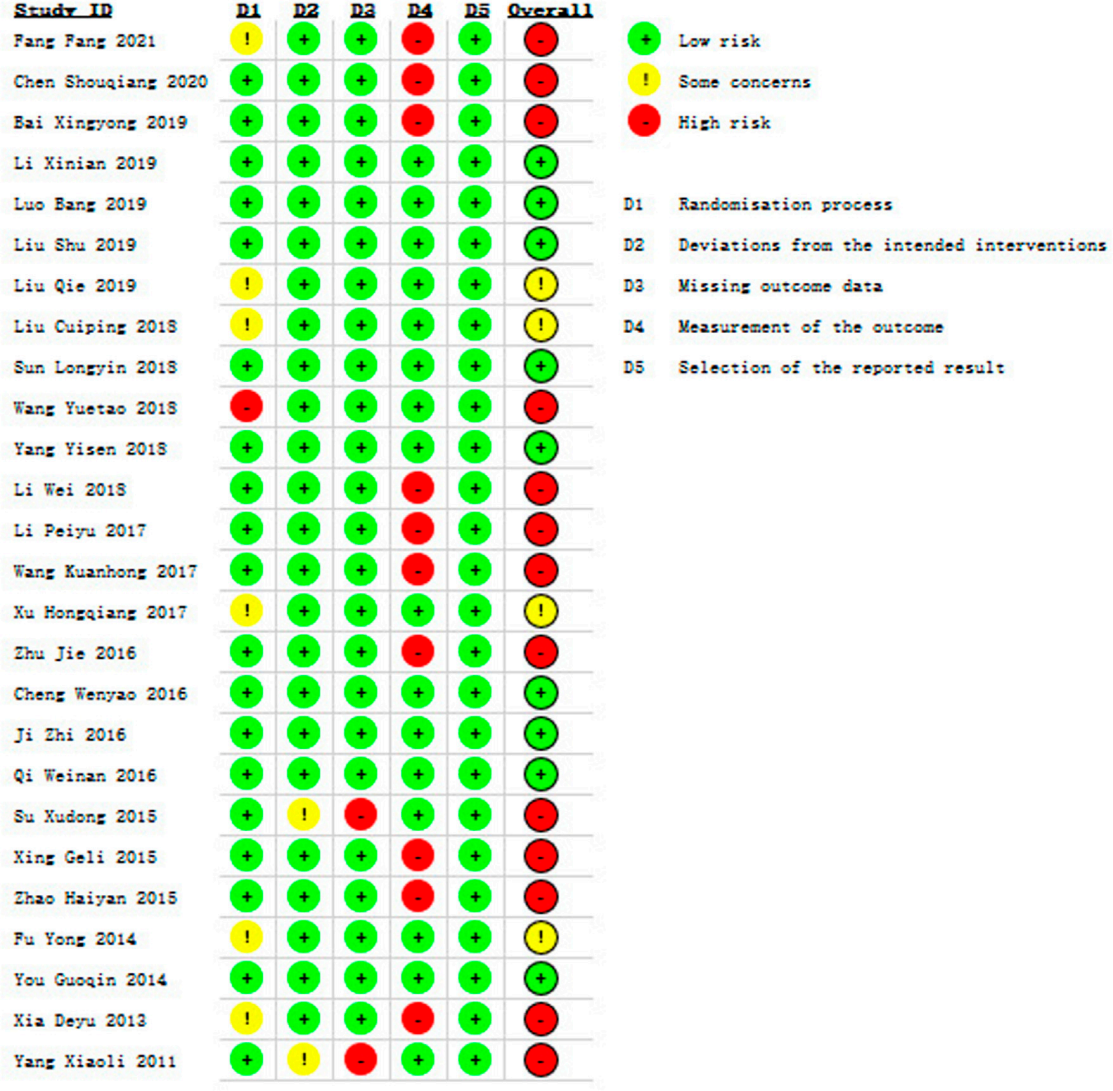
Risk of bias assessment.

In terms of bias, the overall risk of bias was identified high in thirteen studies (Fang, 2021; Chen et al., 2020; Bai, 2019; Wang, 2018; Li et al., 2018; Li, 2017; Wang, 2017; Zhu et al., 2016; Su et al., 2015; Xing and Abudushalamu, 2015; Zhao, 2015; Xia, 2013; Yang, 2011), moderate in four studies (Liu, 2019; Liu et al., 2018; Xu H et al., 2017; Fu, 2014) and low in nine studies (Li X et al., 2019; Luo, 2019; Liu et al., 2019; Sun, 2018; Yang, 2018; Cheng et al., 2016; Ji et al., 2016; Qi et al., 2016; You, 2014), which is shown in Figure 2 and Supplementary Figure S1. We presented some concerns in the randomization process, as 6 studies (Xia, 2013; Fu, 2014; Xu H et al., 2017; Liu et al., 2018; Liu, 2019; Fang, 2021) provided insufficient information in the method of random allocation, and 1 study (Wang, 2018) showed high risk due to the use of a semirandomization method. The risk of bias in the deviations from intended interventions of two studies (Yang, 2011; Su et al., 2015) were considered as some concerns due to the use of per-protocol analysis for their descriptions of shedding cases were not included in statistics, and these two studies were rated as high risk for missing outcome data because the proportions of losing participants were directly linked to the subsequent symptoms in stroke patients. The risk of bias from measurement of the outcome in ten studies (Xia, 2013; Xing and Abudushalamu, 2015; Zhao, 2015; Zhu et al., 2016; Li, 2017; Wang, 2017; Li et al., 2018; Bai, 2019; Chen et al., 2020; Fang, 2021) were evaluated as high risk owing to the use of self-design methods to measure clinical efficacy rate.

### 3.4 Cognitive Function Assessment

#### 3.4.1 Montreal Cognitive Assessment

**FIGURE 3:**
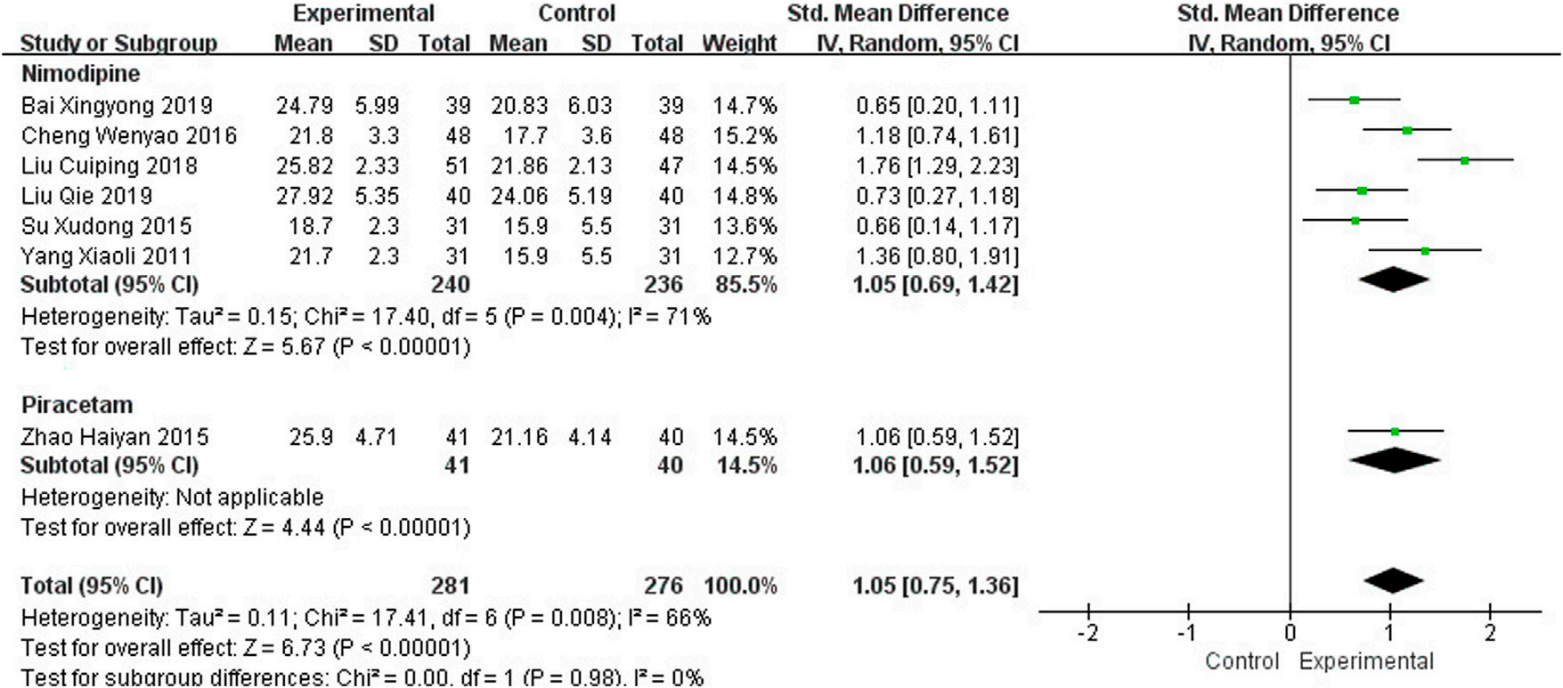
Meta-analysis of MoCA by NBP as monotherapy.

Seven studies, including 557 participants, reported the clinical efficacy of NBP as monotherapy, which is shown in Figure 3. Subgroup analysis was performed according to different intervention measures in the control group. Six studies (Yang, 2011; Su et al., 2015; Cheng et al., 2016; Liu et al., 2018; Bai, 2019; Liu, 2019) were treated with Nimodipine (*I* ^2^ = 71%, *p* = 0.004), and the control group in 1 study (Zhao, 2015) was treated with Piracetam. A random effects model was used for the meta-analysis, showing a significant benefit in favor of NBP as monotherapy in MoCA scores (SMD_N_ = 1.05, 95% CI [0.69, 1.42], *p* < 0.00001; SMD_P_ = 1.06, 95% CI [0.59, 1.52], *p* < 0.00001).

**FIGURE 4:**
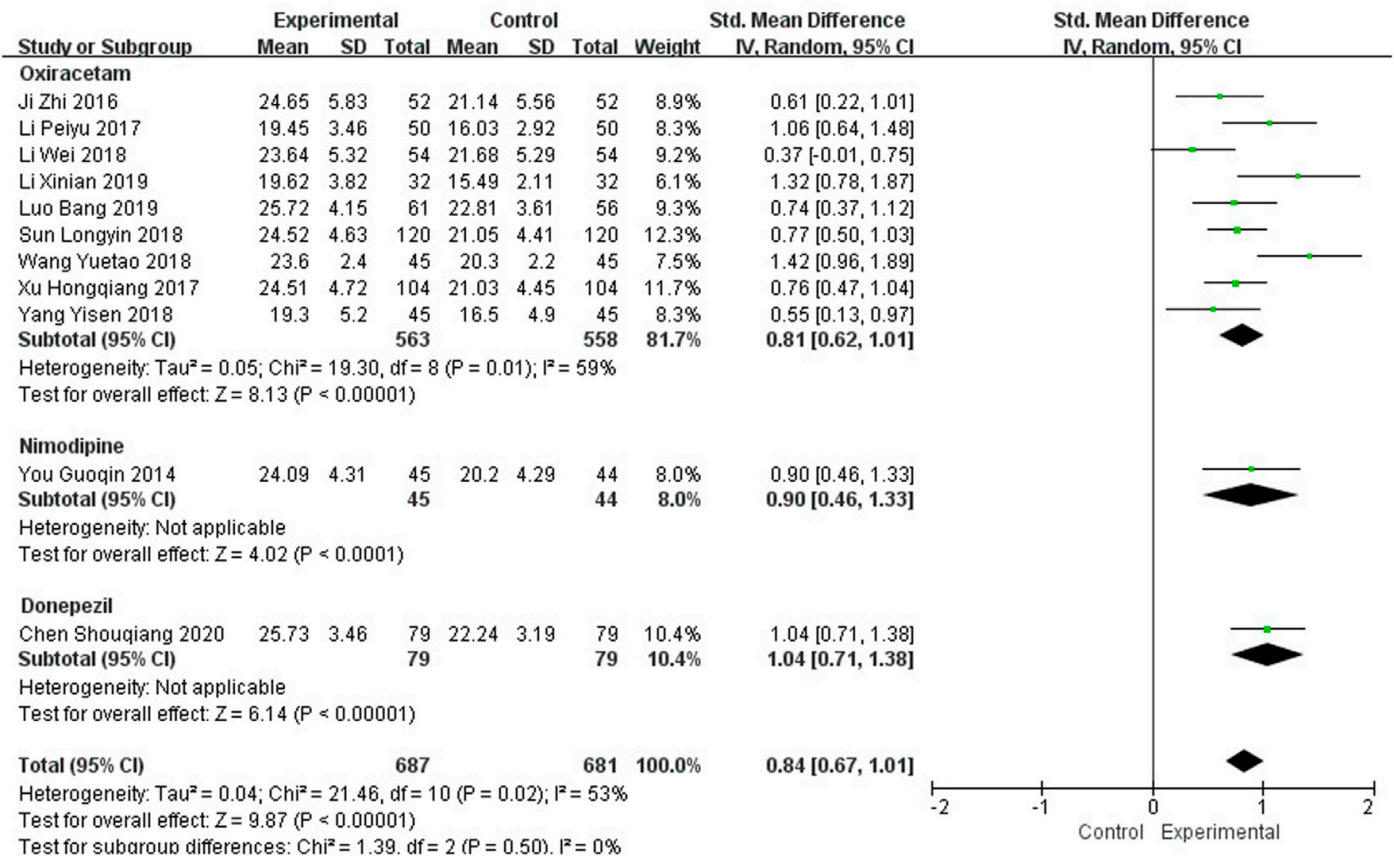
Meta-analysis of MoCA by NBP as combination therapy.

Eleven studies, including 1,368 participants, reported the clinical efficacy of NBP as combination therapy, which is shown in Figure 4. Subgroup analysis was conducted according to different intervention measures. The control group in 9 studies (Ji et al., 2016; Li, 2017; Xu H et al., 2017; Li et al., 2018; Sun, 2018; Wang, 2018; Yang, 2018; Li X et al., 2019; Luo, 2019) was treated with Oxiracetam (*I* ^2^ = 59%, *p* = 0.01), and 2 studies were treated with Nimodipine (You, 2014) and Donepezil (Chen et al., 2020). We used a random effects model for the meta-analysis and found a statistically significant benefit of NBP as combination therapy in MoCA scores (SMD_O_ = 0.81, 95% CI [0.62, 1.01], *p* < 0.00001; SMD_N_ = 0.90, 95% CI [0.46, 1.33], *p* < 0.0001; SMD_D_ = 1.04, 95% CI [0.71, 1.38], *p* < 0.00001).

#### 3.4.2 Mini-Mental State Examination

**FIGURE 5:**
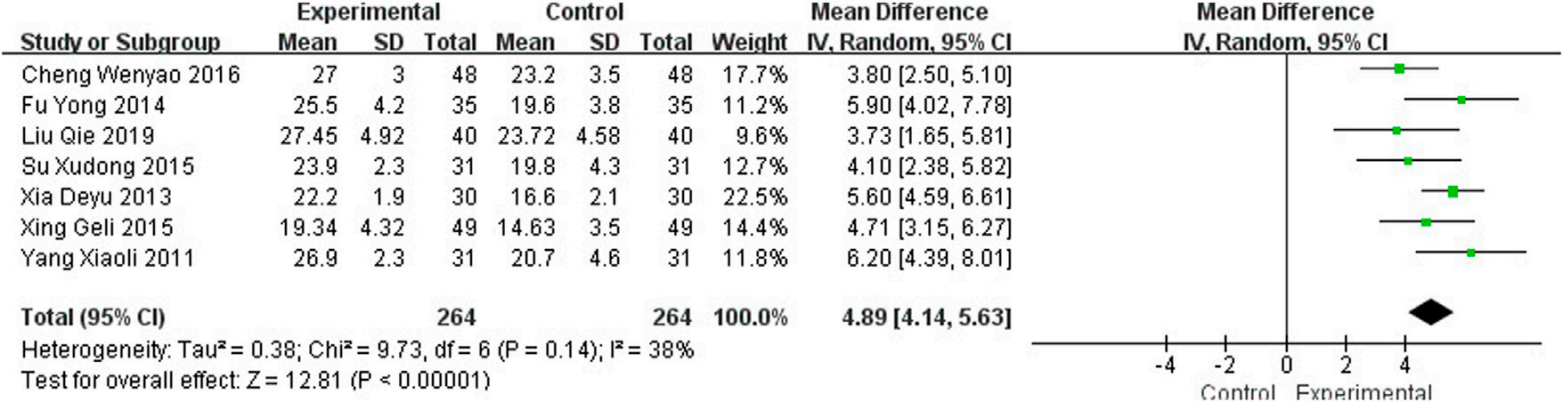
Meta-analysis of MMSE by NBP as monotherapy.

Seven studies (Yang, 2011; Xia, 2013; Fu, 2014; Su et al., 2015; Xing and Abudushalamu, 2015; Cheng et al., 2016; Liu, 2019), including 528 participants, reported the clinical efficacy of NBP as monotherapy, which is shown in Figure 5. The control groups were all treated with Nimodipine, with no obvious heterogeneity (*I* ^2^ = 38%, *p* = 0.14). The meta-analysis using a random effects model showed an important benefit in favor of NBP as monotherapy in MMSE scores (MD_N_ = 4.89, 95% CI [4.14, 5.63], *p* < 0.00001).

**FIGURE 6:**
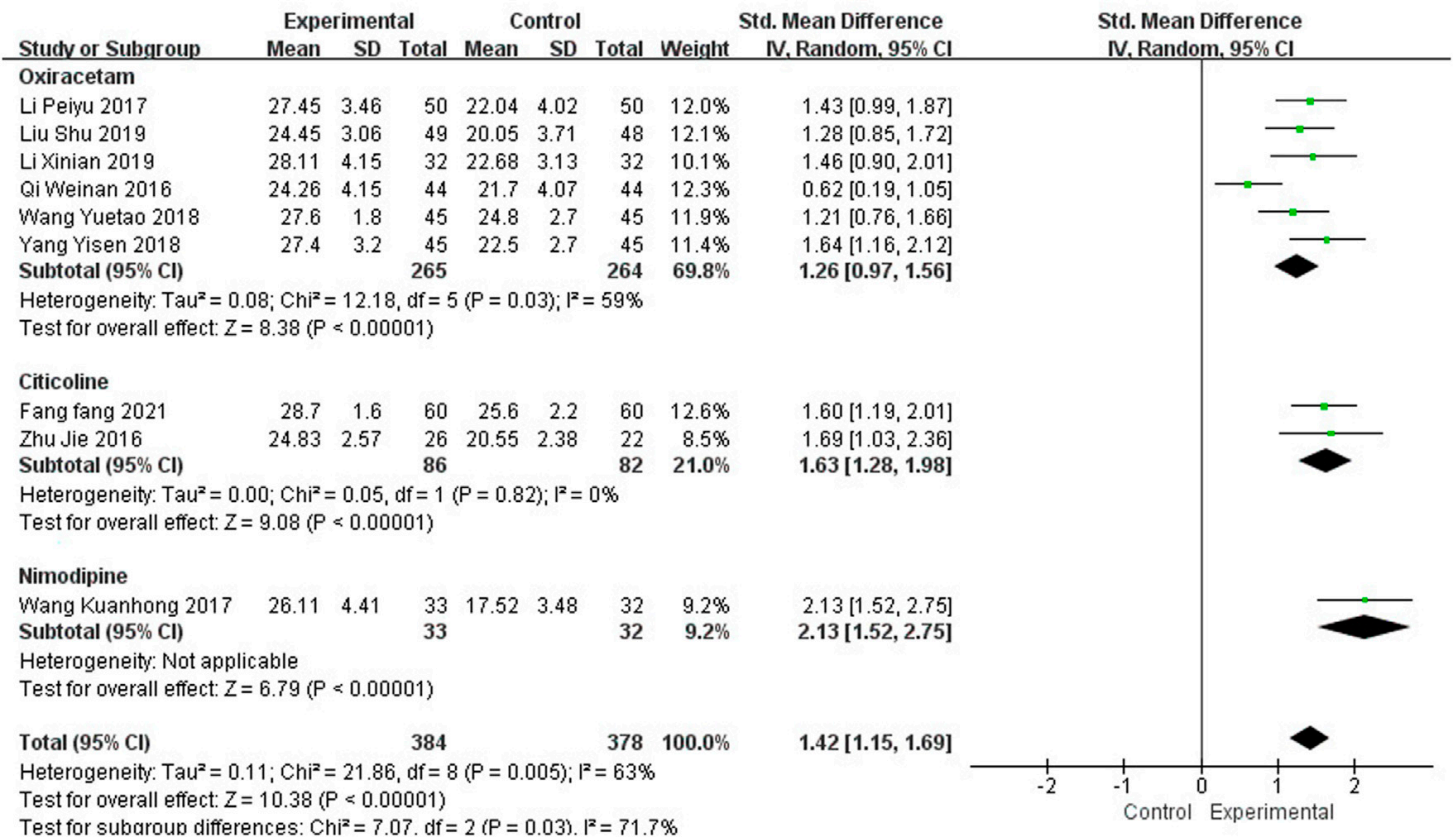
Meta-analysis of MMSE by NBP as combination therapy.

Nine studies, including 762 participants, reported the clinical efficacy of NBP as combination therapy, which is shown in Figure 6. Subgroup analysis was performed according to different intervention measures. The control group in 6 studies (Qi et al., 2016; Li, 2017; Wang, 2018; Yang, 2018; Li X et al., 2019; Liu et al., 2019) was treated with Oxiracetam (*I* ^2^ = 59%, *p* = 0.03), 2 studies (Zhu et al., 2016; Fang, 2021) was treated with Citicoline (*I* ^2^ = 0%, *p* = 0.82), and 1 study (Wang, 2017) was treated with Nimodipine. A random effects model was used for the meta-analysis, the results indicated significant benefit in favor of NBP as combination therapy in MMSE scores (SMD_O_ = 1.26, 95% CI [0.97, 1.56], *p* < 0.00001; SMD_C_ = 1.63, 95% CI [1.28, 1.98], *p* < 0.00001; SMD_N_ = 2.13, 95% CI [1.52, 2.75], *p* < 0.00001).

### 3.5 Abilities of Daily Living

#### 3.5.1 Barthel Index

**FIGURE 7:**
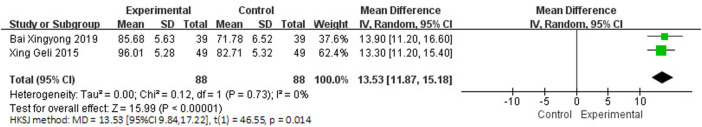
Meta-analysis of BI by NBP as monotherapy.

Three studies involving 238 participants reported the clinical efficacy of NBP as monotherapy. Among them, the score criteria of 1 study (Su et al., 2015) were not clearly described, so it was excluded from data analysis. The control groups of the remaining 2 studies (Xing and Abudushalamu, 2015; Bai, 2019) were all treated with Nimodipine, with no obvious heterogeneity (*I* ^2^ = 0%, *p* = 0.73), which is shown in Figure 7. We used a random effects model for the meta-analysis, and the results showed a statistically significant benefit of NBP as monotherapy in BI scores (MD_N_ = 13.53, HKSJ 95% CI [9.84, 17.22], *p* = 0.014).

**FIGURE 8:**
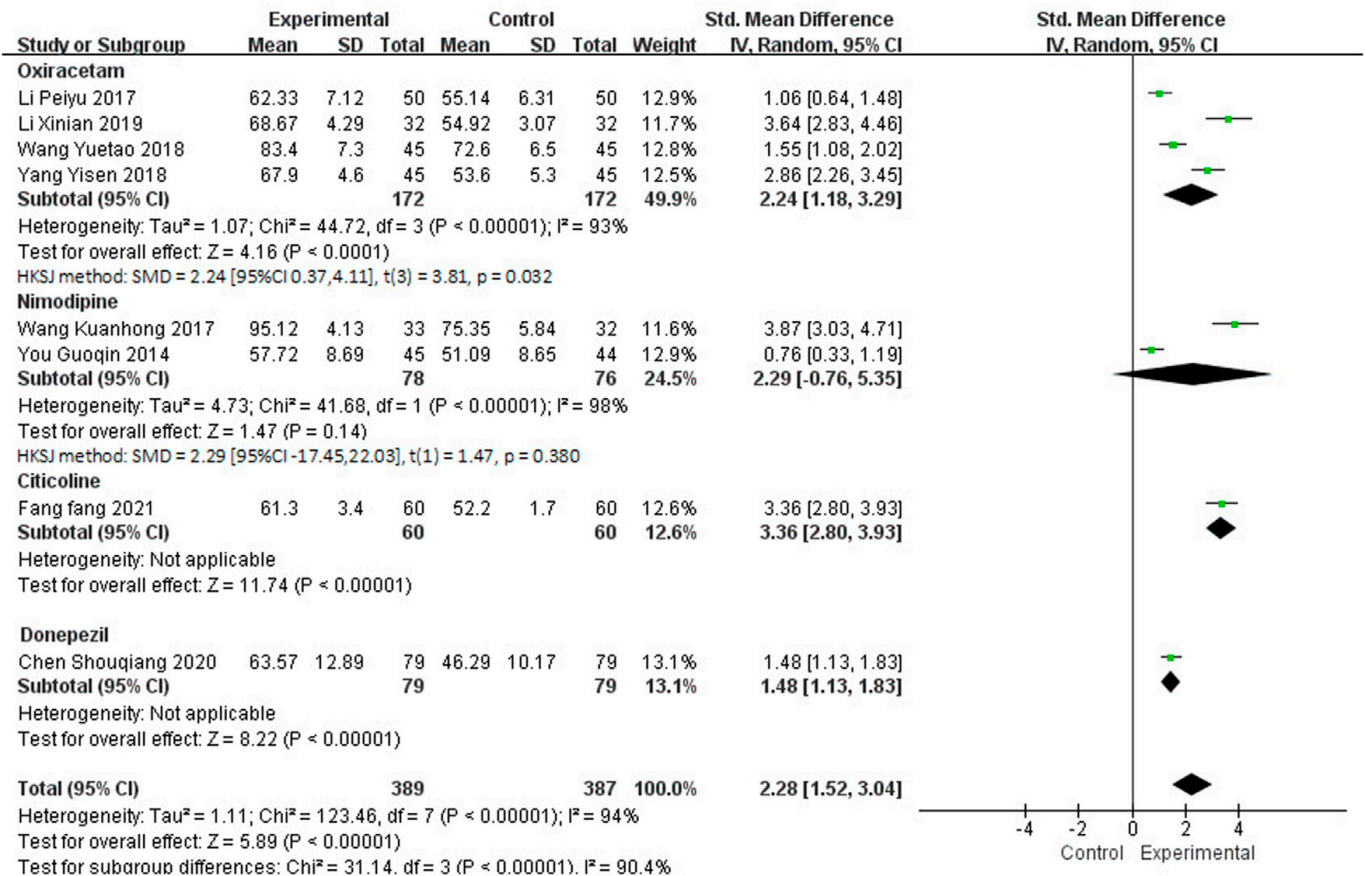
Meta-analysis of BI by NBP as combination therapy.

Twelve studies, including 1,126 participants, reported the clinical efficacy of NBP as combination therapy. Among them, the score criteria of 4 studies (Luo, 2019; Liu et al., 2019; Zhu et al., 2016; Qi et al., 2016) were not clearly described, so they were excluded from data analysis. Subgroup analysis was performed according to the different intervention measures of the remaining 8 studies, which is shown in Figure 8. The control group in 4 studies (Li, 2017; Wang, 2018; Yang, 2018; Li X et al., 2019) was treated with Oxiracetam (*I* ^2^ = 93%, *p* < 0.00001), 2 studies (You, 2014; Wang, 2017) was treated with Nimodipine (*I* ^2^ = 98%, *p* < 0.00001), and 2 studies was treated with Citicoline (Fang, 2021) and Donepezil (Chen et al., 2020). A random effects model was used for meta-analysis, showing an important benefit in favor of NBP as combination therapy in BI scores in Oxiracetam, Citicoline and Donepezil group (SMD_O_ = 2.24, HKSJ 95%CI [0.37, 4.11], *p* = 0.032; SMD_C_ = 3.36, 95%CI [2.80, 3.93], *p* < 0.00001; SMD_D_ = 1.48, 95%CI [1.13, 1.83], *p* < 0.00001), and no significant benefit in Nimodipine group (SMD_N_ = 2.29, HKSJ 95%CI [−17.45, 22.03], *p* = 0.380).

#### 3.5.2 Activities of Daily Living Scale

**FIGURE 9:**
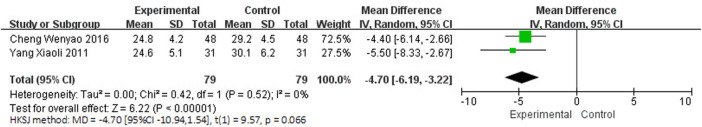
Meta-analysis of ADL by NBP as monotherapy.

Two studies (Yang, 2011; Cheng et al., 2016), involving 158 participants, reported the clinical efficacy of NBP as monotherapy, which is shown in Figure 9. The control groups were all treated with Nimodipine, with no obvious heterogeneity (*I* ^2^ = 0%, *p* = 0.52). The meta-analysis using a random effects model suggested no significant benefit in favor of NBP as monotherapy in ADL scores (MD_N_ = −4.70, HKSJ 95% CI [−10.94, 1.54], *p* = 0.066).

### 3.6 Neurological Deficit

#### 3.6.1 National Institutes of Health Stroke Scale

One study (Bai, 2019) reported the clinical efficacy of NBP as monotherapy, including 78 participants. The control group was treated with Nimodipin. The results of the meta-analysis showed a beneficial effect of NBP as monotherapy on NIHSS scores (MD_N_ = −3.86, 95% CI [−5.22, −2.50], *p* < 0.00001).

**FIGURE 10:**
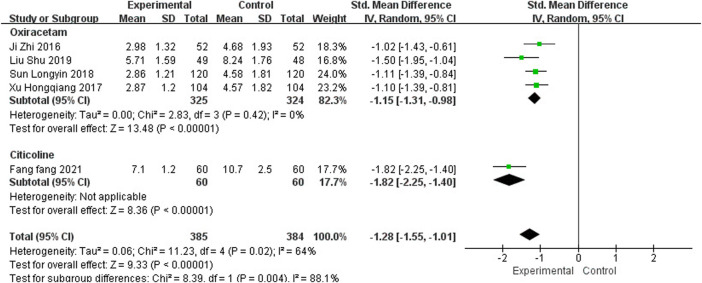
Meta-analysis of NIHSS by NBP as combination therapy.

Five studies, including 769 participants, reported the clinical efficacy of NBP as combination therapy, which is shown in Figure 10. Subgroup analysis was performed according to different intervention measures. Four studies (Ji et al., 2016; Xu H et al., 2017; Sun, 2018; Liu et al., 2019) were treated with Oxiracetam (*I* ^2^ = 0%, *p* = 0.42), and the control group in 1 study (Fang, 2021) was treated with Citicoline. We used a random effects model for the meta-analysis and found that NBP as combination therapy had a statistically significant effect on improving NIHSS scores (SMD_O_ = −1.15, 95% CI [−1.31, −0.98], *p* < 0.00001; SMD_C_ = −1.82, 95% CI [−2.25, −1.40], *p* < 0.00001).

#### 3.6.2 China Stroke Scale

One study (Li et al., 2018) reported the clinical efficacy of NBP as combination therapy, including 108 participants. The control group was treated with Oxiracetam, and the results of the meta-analysis showed a significant effect of NBP as combination therapy on CSS scores (MD_O_ = −7.11, 95% CI [−8.42, −5.80], *p* < 0.00001).

### 3.7 Adverse Events

**FIGURE 11:**
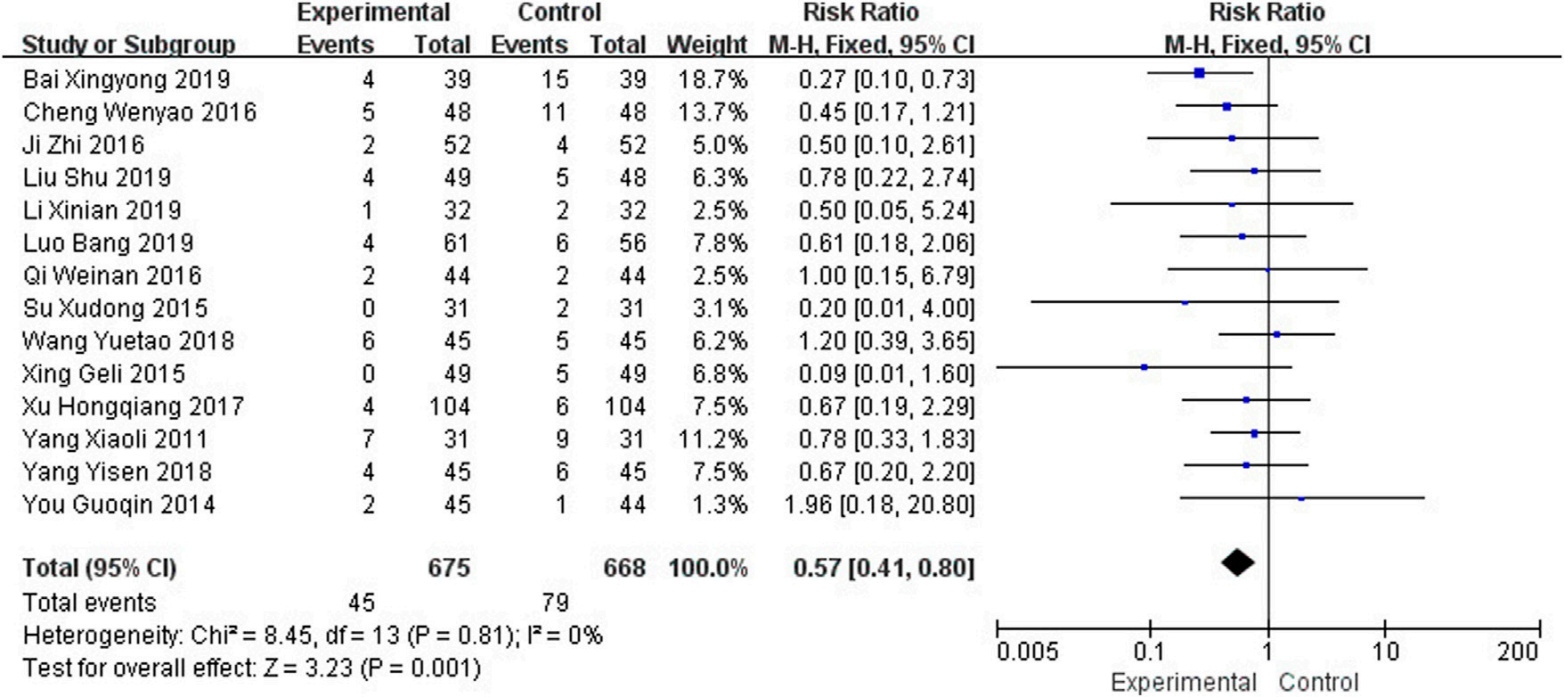
Meta-analysis of adverse events.

Seventeen studies reported the occurrence of adverse events, among which 3 studies (Xia, 2013; Zhao, 2015; Li et al., 2018) reported no adverse reactions in the intervention and control groups, while the other 14 studies (Yang, 2011; You, 2014; Su et al., 2015; Xing and Abudushalamu, 2015; Cheng et al., 2016; Ji et al., 2016; Qi et al., 2016; Xu H et al., 2017; Wang, 2018; Yang, 2018; Bai, 2019; Li X et al., 2019; Liu et al., 2019; Luo, 2019) reported that most adverse reactions were elevated transaminase, gastrointestinal discomfort, nausea and vomiting, anorexia, dizziness, insomnia, and decreased blood pressure. The heterogeneity was not obvious (*I* ^2^ = 0%, *p* = 0.81), and a fixed effects model was used for meta-analysis, as shown in Figure 11, and the results showed that the number of adverse events in the intervention group was lower than that in the control group (RR = 0.57, 95% CI [0.41, 0.80], *p* = 0.001).

### 3.8 Publication Bias

**FIGURE 12:**
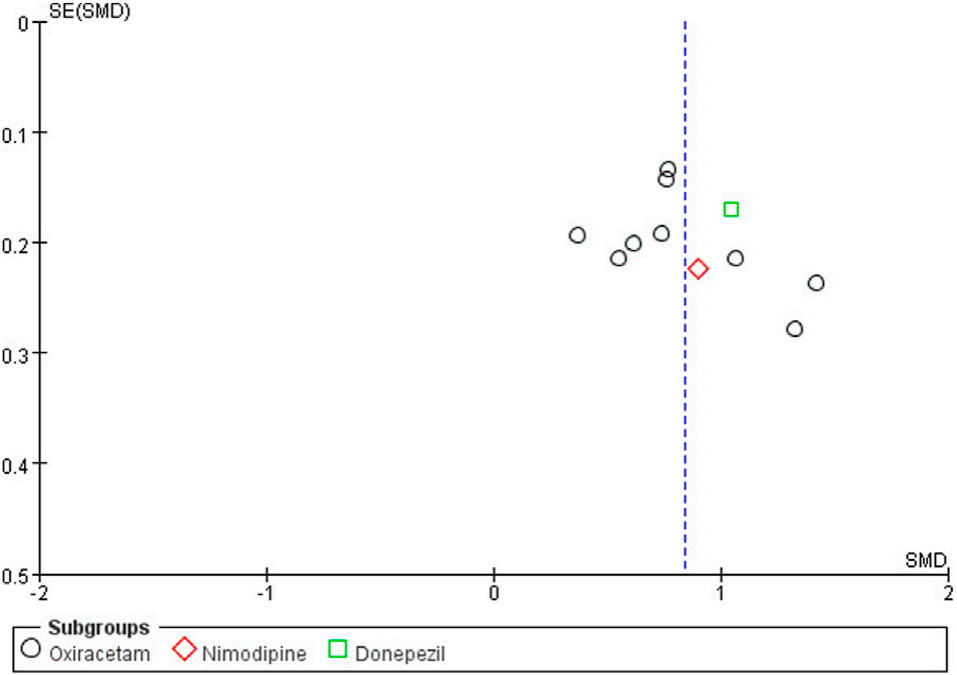
Funnel plot of the MoCA score of NBP as combination therapy.

The funnel plot was drawn on the MoCA score of NBP as combination therapy, as shown in Figure 12. It was found that the graph was slightly biased due to the asymmetry of left and right scatter points, suggesting the possibility of publication bias.

### 3.9 GRADE Assessment

GRADE system was used to evaluate the overall evidence of the above six outcomes, as is shown in Tables 2, 3. The certainty of the evidence indicated low or very low with serious methodological problems, obvious inter-study heterogeneity and significant publication bias.

**TABLE 2:** Certainty assessment of evidence according to GRADE (NBP as monotherapy).

Outcomes	Risk of bias	Inconsistency	Indirectness	Imprecision	Publication bias	No. of patients (studies)	Absolute effects (95% CI)	Certainty of the evidence
MoCA	serious^a^	serious^b^	not serious	not serious	strongly suspected^c^	557 (7)	SMD 1.05 higher (0.75 higher to 1.36 higher)	⊕ ◯ ◯ ◯ Very low
MMSE	serious^a^	not serious	not serious	not serious	strongly suspected^c^	528 (7)	MD 4.89 higher (4.14 higher to 5.63 higher)	⊕⊕ ◯ ◯ Low
BI	very serious^d^	not serious	not serious	not serious	strongly suspected^c^	176 (2)	MD 13.53 higher (11.87 higher to 15.18 higher)	⊕ ◯ ◯ ◯ Very low
ADL	serious^a^	not serious	not serious	not serious	strongly suspected^c^	158 (2)	MD 4.7 lower (6.19 lower to 3.22 lower)	⊕⊕ ◯ ◯ Low
NIHSS	very serious^d^	serious^e^	not serious	serious^f^	strongly suspected^c^	78 (1)	MD 3.86 lower (5.22 lower to 2.50 lower)	⊕ ◯ ◯ ◯ Very low

Grade assessment with justification given as follows:

a Most studies are at high RoB.

b 50% < *I* ^2^ < 75%.

c Too few studies.

d All studies are at high RoB.

e Not possible to determine.

f Small simple size.

**TABLE 3:** Certainty assessment of evidence according to GRADE (NBP as combination therapy).

Outcomes	Risk of bias	Inconsistency	Indirectness	Imprecision	Publication bias	No. of patients (studies)	Absolute effects (95% CI)	Certainty of the evidence
MoCA	not serious	serious^a^	not serious	not serious	strongly suspected^b^	1,368 (11)	SMD 0.84 higher (0.67 higher to 1.01 higher)	⊕⊕ ◯ ◯ Low
MMSE	serious^c^	serious^a^	not serious	not serious	strongly suspected^d^	762 (9)	SMD 1.42 higher (1.15 higher to 1.69 higher)	⊕ ◯ ◯ ◯ Very low
BI	serious^c^	very serious^e^	not serious	not serious	strongly suspected^d^	776 (8)	SMD 2.28 higher (1.52 higher to 3.04 higher)	⊕ ◯ ◯ ◯ Very low
NIHSS	not serious	serious^a^	not serious	not serious	strongly suspected^d^	769 (5)	SMD 1.28 lower (1.55 lower to 1.01 lower)	⊕⊕ ◯ ◯ Low
CSS	very serious^f^	serious^g^	not serious	serious^h^	strongly suspected^d^	108 (1)	MD 7.11 lower (8.42 lower to 5.80 lower)	⊕ ◯ ◯ ◯ Very low

Grade assessment with justification given as follows:

a 50% < *I* ^2^ < 75%.

b Based on the publication bias test, there is apparent asymmetry in the funnel plot.

c Most studies are at high RoB.

d Too few studies.

e *I* ^2^ ≥ 75%.

f All studies are at high RoB.

g Not possible to determine.

h Small simple size.

## 4 Discussion

### 4.1 Summary of Evidence

A total of 26 randomized controlled trials were included in our systematic review, including 2,571 patients suffering from PSCI. Through subgroup analysis of different intervention measures in the control group, we found that NBP as monotherapy could effectively improve the recovery of cognitive function, promote the ability of daily living, and reduce neurological deficits. In addition, the combination therapy also had a corresponding synergistic effect on PSCI patients, which is basically consistent with previous research conclusions (Feng et al., 2018). Fourteen out of 26 studies reported adverse reactions during the trial, most of which were elevated transaminase and gastrointestinal discomfort, suggesting that NBP was better tolerated in patients with PSCI.

### 4.2 Implications for Practice

The use of NBP for PSCI patients has increased in the past few decades. However, there is still limited evidence on the use of drug therapy for the treatment of PSCI. The available evidence from the present study indicated, to a certain extent, that NBP has a positive effect on the cognitive function of patients with PSCI, which can be considered for clinical application in the future.

### 4.3 Implications for Research

At present, there is still a lack of consistent guideline recommendations for PSCI therapeutic schedules; thus, drug treatment projects for cognitive function after stroke are a significant and difficult point in clinical practice. In our systematic review, we evaluated the efficacy and safety of NBP in PSCI patients and proposed some recommendations for future researchers.

First, current studies have indicated that PSCI generally affects complex cognitive function in multiple domains (Middleton et al., 2014), and assessment scales used only for cognitive screening tests such as MoCA and MMSE are insensitive in detecting cognitive function after stroke (Jokinen et al., 2015). Some studies demonstrated that ADAS-Cog has certain clinical value for cognitive recovery of vascular factors (Rockwood et al., 2017), but it still lacks specificity (Kueper et al., 2018). Future studies should pay more attention to the score results of VADAS-Cog to provide better detection of vascular conditions (Ylikoski et al., 2007).

Second, cognitive impairment has a continuous dynamic change trend after stroke (Levine et al., 2015). Previous studies confirmed that even during adequate drug treatment, cognitive dysfunction was highly associated with stroke recurrence (Kwon et al., 2020); therefore, the recovery of cognitive function may help control the progression of ischemic stroke. However, only one of the included studies (Yang, 2011) described the recurrence of cerebral infarction after the trial. Endpoint criteria, such as dementia transformation, stroke recurrence and death, deserve further investigation to clarify the clinical efficacy of NBP.

Third, our systematic review suggested a better safety of NBP. However, some eligible studies reported transient elevation of transaminase after NBP administration, which was demonstrated in a systematic review of ischemic stroke (Xu et al., 2019). Therefore, we recommend that future researchers regularly monitor the liver function of patients during treatment and weigh whether NBP can be used in patients with pre-existing liver function damage.

Fourth, effective therapies for ischemic stroke depend on timely restoration of blood supply to brain tissue (Wu et al., 2019); therefore, early identification of symptoms may be beneficial to rehabilitation. The specification of NBP included patients with acute stroke less than 72 h after onset, while none of the included studies stated the intervention time of NBP, and whether timely treatment is beneficial to early recovery of cognitive function deserves further study.

**FIGURE 13:**
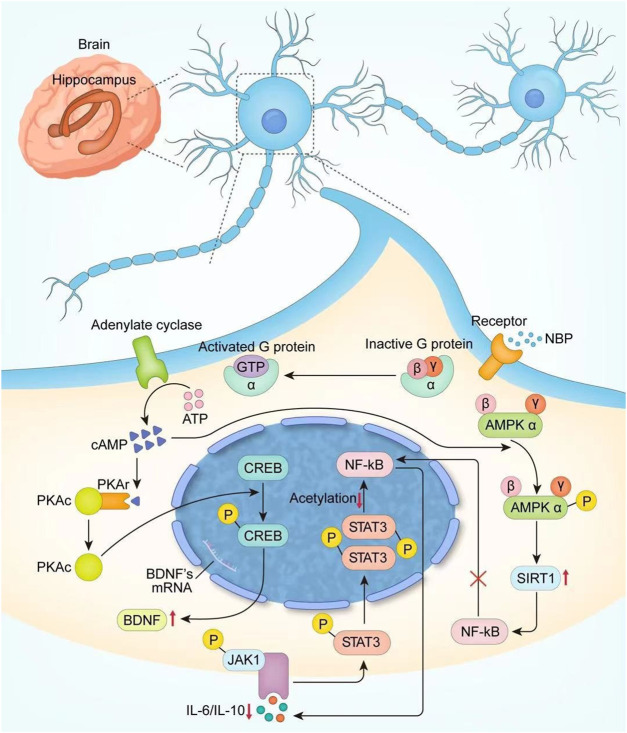
The potential mechanism of NBP.

Finally, NBP is a synthetic compound isolated from the seeds of Apium graveolens (Wang et al., 2018), and a series of clinical trials confirmed the positive effect of it in PSCI patients. We systematically retrieved the possible mechanisms of NBP and summarized them according to the timeline in Table 4, and conducted a more detailed analysis and localization of NBP in the light of neuroplasticity (On the one hand, NBP can increase the newborn neuron survival rate by increasing CREB activity and upregulating BDNF expression; on the other hand, it can promote regeneration and repair of myelin sheath by upregulating AMPK/SIRT1 and downregulating the STAT3/NF-κB pathway), which is shown in Figure 13. Although neuroplasticity is a hot spot in current research, the mechanism of NBP has not been completely clarified. We believe that the clinical treatment of NBP is critical in terms of neuroplasticity and suggest that future studies explore the relationship between them to obtain a clear understanding of the potential therapeutic mechanism. In addition, the specification of NBP only focuses on the use of ischemic stroke, and further research is needed to determine whether NBP should be used more widely.

**TABLE 4:** Possible therapeutic mechanisms of NBP.

Published year	Title	Potential mechanisms	Experimental models used
2012	L-3-n-butylphthalide improves cognitive deficits in rats with chronic cerebral ischemia Xu et al. (2012)	(1) Reduced long-term potentiation (LTP) decreasing in the hippocampus (2) Attenuated glial fibrillary acidic protein (GFAP)-positive astrocytes	Rats, two-vessel occlusion (2-VO)
2015	L-3-n-butylphthalide Promotes Neurogenesis and Neuroplasticity in Cerebral Ischemic Rats Yang et al. (2015)	(1) Promoted the proliferation, survival and differentiation of newborn neural cells (2) Markedly upregulated the expressions of both growth-associated protein-43 and synaptophysin (3) Significantly increased the levels of catalytical subunit of protein kinase A (PKA), protein kinase B (Akt), and cAMP response elementbinding protein (CREB) (4) Obviously inhibited the activation of the signal transducer and activation of transcription 3 (STAT3) and the expressions of cleaved caspase-3 and Bax	Rats, middle cerebral artery occlusion (MCAO)
2017	Dl-3-n-Butylphthalide Treatment Enhances Hemodynamics and Ameliorates Memory Deficits in Rats with Chronic Cerebral Hypoperfusion Xiong et al. (2017)	(1) Significantly elevated cerebral blood flow (CBF) level in the hippocampus and returned it to a normal level at 2 weeks (2) Markedly reduced reactive astrogliosis and cell apoptosis	Rats, bilateral common carotid artery occlusion (BCCAO)
2019	Dl-3-n-Butylphthalide Reduces Cognitive Impairment Induced by Chronic Cerebral Hypoperfusion Through GDNF/GFRα1/Ret Signaling Preventing Hippocampal Neuron Apoptosis Li W et al. (2019)	(1) Reduced hippocampal neuron apoptosis by regulating neuroprotective mechanisms of Glial cell line-derived neurotrophic factor (GDNF), GDNF family receptor alpha-1 (GFRα1), and receptor tyrosine kinase (Ret) signaling	Rats, bilateral common carotid artery occlusion (BCCAO)
2019	dl-3-n-butylphthalide preserves white matter integrity and alleviates cognitive impairment in mice with chronic cerebral hypoperfusion Han et al. (2019)	(1) Protected endothelial cells against oxidative injury, reduced cerebral edema, and maintained the blood–brain barrier (BBB) integrity (2) Effectively decreased the number of activated astrocytes and pro-inflammatory cytokines, as well as the production of MMPs	Rats, bilateral common carotid artery stenosis (BCAS)
2020	Dl-3-n-Butylphthalide Promotes Remyelination and Suppresses Inflammation by Regulating AMPK/SIRT1 and STAT3/NF-κB Signaling in Chronic Cerebral Hypoperfusion Li M et al. (2020)	(1) Alleviated cognitive impairment by promoting remyelination and suppressing inflammation *via* modulation of AMP-activated protein kinase (AMPK)/Sirtuin (SIRT)1 and Signal transducer and activator of transcription (STAT)3/nuclear factor (NF)-κB signaling	Rats, two-vessel occlusion (2-VO)
2020	Dl-3-n-Butylphthalide Alleviates Hippocampal Neuron Damage in Chronic Cerebral Hypoperfusion *via* Regulation of the CNTF/CNTFRα/JAK2/STAT3 Signaling Pathways Li W et al. (2020)	(1) Inhibited the Ciliary neurotrophic factor (CNTF)/Ciliary neurotrophic factor receptor alpha (CNTFRα) expression and activated the Janus kinases 2 (JAK2)/STAT3 pathway to reduce hippocampal neuronal apoptosis	Rats, bilateral common carotid artery occlusion (BCCAO)

### 4.4 Limitations

The findings of our meta-analysis provided evidence to support the clinical efficacy of NBP, but there are some limitations that can be discussed in future studies. First, the methodological quality of the eligible studies is generally low. Although we adopted a rigorous and reproducible method to retrieve and screen the literature, all studies included in this research were conducted in China, raising the question of generalizability to Western patients. Most studies lack information on random allocation, blind design and complete study data, which, to a certain extent, affect the results of intervention effectiveness (Bilandzic et al., 2016; Di Girolamo et al., 2018). Second, clinical heterogeneity is inevitable. The outcome indicators of our meta-analysis were mainly evaluated by assessment scales. However, because of the scale version and subjectivity of the scale scores, heterogeneity between studies could not be further reduced by sensitivity or subgroup analysis. Third, the treatment intervention time was unclear. NBP is generally used for 1–3 months in clinical practice depending on the severity of the disease. However, some high-quality clinical studies set the treatment time as 6 months (Jia et al., 2016), and 5 out of 26 included studies concluded that a treatment time up to 6 months can have a better clinical effect, while none of them described the follow-ups. Fourth, the comorbid conditions of patients was unclear. Stroke usually associated with multiple risk factors and causes. Primary stroke prevention with emphasis on underlying diseases improvement plays an important role in reducing the burden of stroke patients (Caprio and Sorond, 2019). However, most of the included studies provided incomplete information, the treatment measures of other diseases was undefined. Fifth, the result of ADL showed no statistically significant difference, and the effect of NBP on the improvement of this respect was unclear. However, due to the small number of included studies and the general lack of description of evaluation criteria, effects analysis could not be carried out, which impaired the value of research.

## 5 Conclusion

NBP maintains a stable pattern in promoting the recovery of cognitive function and abilities of daily living, as well as reducing neurological deficits in the treatment of PSCI. However, due to the low quality of the methodology, large samples, multicenter and high-quality randomized controlled trials are still needed to verify the clinical efficacy and safety of NBP.

## Data Availability

The original contributions presented in the study are included in the article/Supplementary Material, further inquiries can be directed to the corresponding authors.
