# Supplementary material for: Efficacy and Safety of DL-3-n-Butylphthalide in the Treatment of Poststroke Cognitive Impairment: A Systematic Review and Meta-Analysis
Source: Front Pharmacol. 2022 Jan 25;12:810297. doi: 10.3389/fphar.2021.810297 (PMC8823901; doi:10.3389/fphar.2021.810297)

**Supplementary Material**

**Table of contents**

[**1 Supplemental tables..................................................................................................3**](#_Toc11490)

**1.1 Table S1 The search strategy........................................................................3**

**1.2 Table S2 The list of excluded reports.........................................................10**

**1.3 Table S3 PRISMA 2020 checklist...............................................................11**

**2 Supplemental figures...............................................................................................13**

**2.1 Figure S1: Risk of bias summary................................................................13**

**1 Supplemental tables**

**1.1 Table S1 The search strategy**

| **The search strategy for PubMed** | |
| --- | --- |
| **Nubmer** | **Search terms** |
| #1 | Stroke[MeSH Terms] |
| #2 | Cerebral HemORrhage[MeSH Terms] |
| #3 | ((((((((((((((Stroke*[Title/Abstract]) OR Cerebrovascular Accident*[Title/Abstract]) OR CVA*[Title/Abstract]) OR Apoplexy, Cerebrovascular[Title/Abstract]) OR Vascular Accident*, Brain[Title/Abstract]) OR Cerebrovascular Stroke*[Title/Abstract]) OR Stroke*, Cerebrovascular[Title/Abstract]) OR Apoplexy[Title/Abstract]) OR Cerebral Stroke*[Title/Abstract]) OR Stroke*, Cerebral[Title/Abstract]) OR Stroke*, Acute[Title/Abstract]) OR Acute Stroke*[Title/Abstract]) OR Cerebrovascular Accident*, Acute[Title/Abstract]) OR Acute Cerebrovascular Accident*[Title/Abstract]) |
| #4 | ((((((((HemORrhage*, Cerebrum[Title/Abstract]) OR Cerebrum HemORrhage*[Title/Abstract]) OR Parenchymal HemORrhage*, Cerebral[Title/Abstract]) OR Intracerebral HemORrhage*[Title/Abstract]) OR HemORrhage*, Intracerebral[Title/Abstract]) OR HemORrhage*, Cerebral[Title/Abstract]) OR Cerebral HemORrhage*[Title/Abstract]) OR Brain HemORrhage*, Cerebral[Title/Abstract]) |
| #5 | #1 OR #2 OR #3 OR #4 |
| #6 | Cognitive Dysfunction[MeSH Terms] |
| #7 | ((((((((((((((((((((((((((((Cognitive Dysfunction[Title/Abstract]) OR Cognitive Dysfunctions[Title/Abstract]) OR Dysfunction, Cognitive[Title/Abstract]) OR Dysfunctions, Cognitive[Title/Abstract]) OR Cognitive Impairments[Title/Abstract]) OR Cognitive Impairment[Title/Abstract]) OR Impairment, Cognitive[Title/Abstract]) OR Impairments, Cognitive[Title/Abstract]) OR Mild Cognitive Impairment[Title/Abstract]) OR Cognitive Impairment, Mild[Title/Abstract]) OR Cognitive Impairments, Mild[Title/Abstract]) OR Impairment, Mild Cognitive[Title/Abstract]) OR Impairments, Mild Cognitive[Title/Abstract]) OR Mild Cognitive Impairments[Title/Abstract]) OR Mild Neurocognitive DisORder[Title/Abstract]) OR DisORder, Mild Neurocognitive[Title/Abstract]) OR DisORders, Mild Neurocognitive[Title/Abstract]) OR Mild Neurocognitive DisORders[Title/Abstract]) OR Neurocognitive DisORder, Mild[Title/Abstract]) OR Neurocognitive DisORders[Title/Abstract]) OR Mild Cognitive Decline[Title/Abstract]) OR Cognitive Declines[Title/Abstract]) OR Decline, Cognitive[Title/Abstract]) OR Declines, Cognitive[Title/Abstract]) OR Mental DeteriORation[Title/Abstract]) OR DeteriORation, Mental[Title/Abstract]) OR DeteriORations, Mental[Title/Abstract]) OR Mental DeteriORations[Title/Abstract]) |
| #8 | Dementia, Vascular[MeSH Terms] |
| #9 | ((((((((((((((((((((((((((((((((((((((((Dementia, Vascular[Title/Abstract]) OR Dementias, Vascular[Title/Abstract]) OR Vascular Dementias[Title/Abstract]) OR Vascular Dementia[Title/Abstract]) OR Vascular Dementia, Acute Onset[Title/Abstract]) OR Acute Onset Vascular Dementia[Title/Abstract]) OR SubcORtical Vascular Dementia[Title/Abstract]) OR Dementia, SubcORtical Vascular[Title/Abstract]) OR Dementias, SubcORtical Vascular[Title/Abstract]) OR SubcORtical Vascular Dementias[Title/Abstract]) OR Vascular Dementia, SubcORtical[Title/Abstract]) OR Vascular Dementias, SubcORtical[Title/Abstract]) OR Arteriosclerotic Dementia[Title/Abstract]) OR Arteriosclerotic Dementias[Title/Abstract]) OR Dementia, Arteriosclerotic[Title/Abstract]) OR Dementias, Arteriosclerotic[Title/Abstract]) OR Binswanger Disease[Title/Abstract]) OR Disease, Binswanger[Title/Abstract]) OR Chronic Progressive SubcORtical Encephalopathy[Title/Abstract]) OR Binswanger Encephalopathy[Title/Abstract]) OR Leukoencephalopathy, SubcORtical[Title/Abstract]) OR Leukoencephalopathies, SubcORtical[Title/Abstract]) OR SubcORtical Leukoencephalopathies[Title/Abstract]) OR Encephalopathy, SubcORtical Arteriosclerotic[Title/Abstract]) OR Binswanger's Disease[Title/Abstract]) OR Binswangers Disease[Title/Abstract]) OR Disease, Binswanger's[Title/Abstract]) OR Encephalopathy, SubcORtical, Chronic Progressive[Title/Abstract]) OR SubcORtical Encephalopathy, Chronic Progressive[Title/Abstract]) OR SubcORtical Leukoencephalopathy[Title/Abstract]) OR SubcORtical Arteriosclerotic Encephalopathy[Title/Abstract]) OR Arteriosclerotic Encephalopathy, SubcORtical[Title/Abstract]) OR Arteriosclerotic Encephalopathies, SubcORtical[Title/Abstract]) OR Encephalopathies, SubcORtical Arteriosclerotic[Title/Abstract]) OR SubcORtical Arteriosclerotic Encephalopathies[Title/Abstract]) OR Encephalopathy, Binswanger's[Title/Abstract]) OR Binswanger's Encephalopathy[Title/Abstract]) OR Encephalopathy, Binswangers[Title/Abstract]) OR Encephalopathy, Binswanger[Title/Abstract]) OR Encephalopathy, Chronic Progressive SubcORtical[Title/Abstract]) |
| #10 | Dementia, Multi-Infarct[MeSH Terms] |
| #11 | ((((((((((((((((((Dementia, Multi-Infarct[Title/Abstract]) OR Dementia, Multi Infarct[Title/Abstract]) OR Dementias, Multi-Infarct[Title/Abstract]) OR Multi-Infarct Dementias[Title/Abstract]) OR Dementia Multi-Infarct[Title/Abstract]) OR Dementia Multi Infarct[Title/Abstract]) OR Dementia Multi-Infarcts[Title/Abstract]) OR Multi-Infarct, Dementia[Title/Abstract]) OR Multi-Infarcts, Dementia[Title/Abstract]) OR Dementia, Multiinfarct[Title/Abstract]) OR Dementias, Multiinfarct[Title/Abstract]) OR Multiinfarct Dementia[Title/Abstract]) OR Multiinfarct Dementias[Title/Abstract]) OR Multi-Infarct Dementia[Title/Abstract]) OR Multi Infarct Dementia[Title/Abstract]) OR Lacunar Dementia[Title/Abstract]) OR Dementia, Lacunar[Title/Abstract]) OR Dementias, Lacunar[Title/Abstract]) OR Lacunar Dementias[Title/Abstract] |
| #12 | (((((((PSCI[Title/Abstract]) OR PSD[Title/Abstract]) OR PSCIND[Title/Abstract]) OR post stroke cognitive impairment[Title/Abstract]) OR cognitive impairment after stroke[Title/Abstract]) OR post stroke cognitive impairment no dementia[Title/Abstract]) OR post stroke dementia[Title/Abstract]) |
| #13 | #6 OR #7 OR #8 OR #9 OR #10 OR #11 OR #12 |
| #14 | (((N-butylphthalide[Title/Abstract]) OR (NBP[Title/Abstract]) OR (Butylphthalide[Title/Abstract]) |
| #15 | #5 AND #13 AND #14 |
| #16 | ((((((((((randomized controlled trial[Publication Type]) OR controlled clinical trial[Publication Type]) OR randomized[Title/Abstract]) OR placebo[Title/Abstract]) OR drug therapy[MeSH Subheading]) OR randomly[Title/Abstract]) OR trial[Title/Abstract]) OR groups[Title/Abstract])) NOT ((animals[MeSH Terms]) NOT humans[MeSH Terms])) |
| #17 | #15 AND #16 |
| **The search strategy for Cochrane library** | |
| **Nubmer** | **Search terms** |
| #1 | MeSH descriptor: [Stroke] explode all trees |
| #2 | (Stroke*):ti,ab,kw OR (Cerebrovascular Accident*):ti,ab,kw OR (CVA*):ti,ab,kw OR (Apoplexy, Cerebrovascular):ti,ab,kw OR (Vascular Accident*, Brain):ti,ab,kw OR (Cerebrovascular Stroke*):ti,ab,kw OR (Stroke*, Cerebrovascular):ti,ab,kw OR (Apoplexy):ti,ab,kw OR (Cerebral Stroke*):ti,ab,kw OR (Stroke*, Cerebral):ti,ab,kw OR (Stroke*, Acute):ti,ab,kw OR (Acute Stroke*):ti,ab,kw OR (Cerebrovascular Accident*, Acute):ti,ab,kw OR (Acute Cerebrovascular Accident*):ti,ab,kw |
| #3 | MeSH descriptor: [Cerebral HemORrhage] explode all trees |
| #4 | (HemORrhage*, Cerebrum):ti,ab,kw OR (Cerebrum HemORrhage*):ti,ab,kw OR (Parenchymal HemORrhage*, Cerebral):ti,ab,kw OR (Intracerebral HemORrhage*):ti,ab,kw OR (HemORrhage*, Intracerebral):ti,ab,kw OR (HemORrhage*, Cerebral):ti,ab,kw OR (Cerebral HemORrhage*):ti,ab,kw OR (Brain HemORrhage*, Cerebral):ti,ab,kw |
| #5 | #1 OR #2 OR #3 OR #4 |
| #6 | MeSH descriptor: [Cognitive Dysfunction] explode all trees |
| #7 | (Dysfunctions, Cognitive):ti,ab,kw OR (Cognitive Impairments):ti,ab,kw OR (Impairments, Cognitive):ti,ab,kw OR (Impairment, Cognitive):ti,ab,kw OR (Dysfunction, Cognitive):ti,ab,kw OR (Cognitive Dysfunctions):ti,ab,kw OR (Cognitive Impairment):ti,ab,kw OR (Mild Neurocognitive DisORders):ti,ab,kw OR (Impairment, Mild Cognitive):ti,ab,kw OR (Neurocognitive DisORders, Mild):ti,ab,kw OR (Mild Cognitive Impairment):ti,ab,kw OR (Impairments, Mild Cognitive):ti,ab,kw OR (DisORder, Mild Neurocognitive):ti,ab,kw OR (Mild Cognitive Impairments):ti,ab,kw OR (Cognitive Impairment, Mild):ti,ab,kw OR (Mild Neurocognitive DisORder):ti,ab,kw OR (DisORders, Mild Neurocognitive):ti,ab,kw OR (Neurocognitive DisORder, Mild):ti,ab,kw OR (Cognitive Impairments, Mild):ti,ab,kw OR (Mental DeteriORations):ti,ab,kw OR (Decline, Cognitive):ti,ab,kw OR (Cognitive Decline):ti,ab,kw OR (Mental DeteriORation):ti,ab,kw OR (DeteriORations, Mental):ti,ab,kw OR (DeteriORation, Mental):ti,ab,kw OR (Declines, Cognitive):ti,ab,kw OR (Cognitive Declines):ti,ab,kw |
| #8 | MeSH descriptor: [Dementia, Vascular] explode all trees |
| #9 | (Vascular Dementias):ti,ab,kw OR (Dementias, Vascular):ti,ab,kw OR (Vascular Dementia):ti,ab,kw OR (Dementia, SubcORtical Vascular):ti,ab,kw OR (Vascular Dementia, SubcORtical):ti,ab,kw OR (Dementias, SubcORtical Vascular):ti,ab,kw OR (SubcORtical Vascular Dementias):ti,ab,kw OR (Vascular Dementias, SubcORtical):ti,ab,kw OR (SubcORtical Vascular Dementia):ti,ab,kw OR (Binswanger's Encephalopathy):ti,ab,kw OR (Binswanger Encephalopathy):ti,ab,kw OR (Chronic Progressive SubcORtical Encephalopathy):ti,ab,kw OR (Encephalopathy, Binswangers):ti,ab,kw OR (Arteriosclerotic Encephalopathies, SubcORtical):ti,ab,kw OR (Disease, Binswanger):ti,ab,kw OR (Arteriosclerotic Encephalopathy, SubcORtical):ti,ab,kw OR (Encephalopathies, SubcORtical Arteriosclerotic):ti,ab,kw OR (SubcORtical Arteriosclerotic Encephalopathies):ti,ab,kw OR (SubcORtical Arteriosclerotic Encephalopathy):ti,ab,kw OR ( Encephalopathy, SubcORtical, Chronic Progressive):ti,ab,kw OR (Leukoencephalopathies, SubcORtical):ti,ab,kw OR (SubcORtical Leukoencephalopathies):ti,ab,kw OR (Encephalopathy, Binswanger's):ti,ab,kw OR (Encephalopathy, Chronic Progressive SubcORtical):ti,ab,kw OR (Disease, Binswanger's):ti,ab,kw OR (Encephalopathy, SubcORtical Arteriosclerotic):ti,ab,kw OR ( Leukoencephalopathy, SubcORtical):ti,ab,kw OR ( SubcORtical Leukoencephalopathy):ti,ab,kw OR (Binswangers Disease):ti,ab,kw OR (SubcORtical Encephalopathy, Chronic Progressive):ti,ab,kw OR (Binswanger's Disease):ti,ab,kw OR (Binswanger Disease):ti,ab,kw OR (Encephalopathy, Binswanger):ti,ab,kw OR (Acute Onset Vascular Dementia):ti,ab,kw OR (Vascular Dementia, Acute Onset):ti,ab,kw OR (Arteriosclerotic Dementias):ti,ab,kw OR (Dementia, Arteriosclerotic):ti,ab,kw OR (Arteriosclerotic Dementia):ti,ab,kw OR (Dementias, Arteriosclerotic):ti,ab,kw |
| #10 | MeSH descriptor: [Dementia, Multi-Infarct] explode all trees |
| #11 | (Multi-Infarct Dementias):ti,ab,kw OR (Multi Infarct Dementia):ti,ab,kw OR (Multi-Infarct, Dementia):ti,ab,kw OR (Multiinfarct Dementia):ti,ab,kw OR (Multi-Infarct Dementia):ti,ab,kw OR (Dementia Multi-Infarcts):ti,ab,kw OR (Dementia, Multi Infarct):ti,ab,kw OR (Dementias, Multi-Infarct):ti,ab,kw OR (Multi-Infarcts, Dementia):ti,ab,kw OR (Dementia, Multiinfarct):ti,ab,kw OR (Dementia Multi-Infarct):ti,ab,kw OR (Dementia Multi Infarct):ti,ab,kw OR (Dementias, Multiinfarct):ti,ab,kw OR (Multiinfarct Dementias):ti,ab,kw OR (Dementia, Lacunar):ti,ab,kw OR (Dementias, Lacunar):ti,ab,kw OR (Lacunar Dementia):ti,ab,kw OR (Lacunar Dementias):ti,ab,kw |
| #12 | (PSCI):ti,ab,kw OR (PSD):ti,ab,kw OR (PSCIND):ti,ab,kw OR (post stroke cognitive impairment):ti,ab,kw OR (cognitive impairment after stroke):ti,ab,kw OR (post stroke cognitive impairment no dementia):ti,ab,kw OR (post stroke dementia):ti,ab,kw |
| #13 | #6 OR #7 OR #8 OR #9 OR #10 OR #11 OR #12 |
| #14 | (N-butylphthalide):ti,ab,kw OR (NBP):ti,ab,kw OR (Butylphthalide):ti,ab,kw |
| #15 | #5 AND #13 AND #14 |
| **The search strategy for Embase** | |
| **Nubmer** | **Search terms** |
| #1 | 'Stroke'/exp |
| #2 | 'Stroke*':ti,ab OR 'Cerebrovascular Accident':ti,ab OR 'CVA*':ti,ab OR 'Apoplexy, Cerebrovascular':ti,ab OR 'Vascular Accident*, Brain':ti,ab OR 'Cerebrovascular Stroke*':ti,ab OR 'Stroke*, Cerebrovascular':ti,ab OR 'Apoplexy':ti,ab OR 'Cerebral Stroke*':ti,ab OR 'Stroke*, Cerebral':ti,ab OR 'Stroke*, Acute':ti,ab OR 'Acute Stroke*':ti,ab OR 'Cerebrovascular Accident*, Acute':ti,ab OR 'Acute Cerebrovascular Accident*':ti,ab |
| #3 | 'Cerebral HemORrhage'/exp |
| #4 | 'HemORrhage*, Cerebrum':ti,ab OR 'Cerebrum HemORrhage*':ti,ab OR 'Parenchymal HemORrhage*, Cerebral':ti,ab OR 'Intracerebral HemORrhage*':ti,ab OR 'HemORrhage*, Intracerebral':ti,ab OR 'HemORrhage*, Cerebral':ti,ab OR 'Cerebral HemORrhage*':ti,ab OR 'Brain HemORrhage*, Cerebral':ti,ab |
| #5 | #1 OR #2 OR #3 OR #4 |
| #6 | 'Vascular Neurocognitive DisORders':ti,ab OR 'Vascular Cognitive DisORders':ti,ab OR 'Vascular Neurocognitive DisORder':ti,ab OR 'Vascular Mild Cognitive Impairment':ti,ab OR 'Post Stroke Cognitive Impairment':ti,ab OR 'Post-Stroke Dementia':ti,ab OR 'SubcORtical Vascular Dementia':ti,ab OR 'Ischemic Vascular Dementia':ti,ab OR 'SubcORtical Vascular Disease':ti,ab OR 'Primary Degenerative Dementia':ti,ab OR 'Mixed Dementia':ti,ab OR 'Cerebrovascular Cognitive Impairment':ti,ab OR 'Multi Infarct Dementia':ti,ab OR 'SubcORtical Ischemic Vascular Disease':ti,ab |
| #7 | 'Arteriosclerotic Encephalopathies, SubcORtical':ti,ab OR 'Encephalopathies, SubcORtical Arteriosclerotic':ti,ab OR 'SubcORtical Arteriosclerotic Encephalopathies':ti,ab OR 'Encephalopathy,Binswangers':ti,ab OR 'Encephalopathy, Binswanger':ti,ab OR 'Encephalopathy, Chronic Progressive SubcORtical':ti,ab OR 'SubcORtical Ischemie Vasculardementia':ti,ab |
| #8 | 'Encephalopathy, SubcORtical, Chronic Progressive':ti,ab OR 'SubcORtical Encephalopathy, Chronic Progressive':ti,ab OR 'SubcORtical Leukoencephalopathy':ti,ab OR 'SubcORtical Arteriosclerotic Encephalopathy':ti,ab OR 'Arteriosclerotic Encephalopathy, SubcORtical':ti,ab |
| #9 | 'Binswangers Disease':ti,ab OR 'SubcORtical Leukoencephalopathies':ti,ab OR 'Encephalopathy, SubcORtical Arteriosclerotic':ti,ab |
| #10 | 'Binswanger Disease':ti,ab OR 'Disease, Binswanger':ti,ab OR 'Chronic Progressive SubcORtical Encephalopathy':ti,ab OR 'Binswanger Encephalopathy':ti,ab OR 'Leukoencephalopathy, SubcORtical':ti,ab OR 'Leukoencephalopathies, SubcORtical':ti,ab OR 'Vascular Dementias, SubcORtical':ti,ab OR 'Arteriosclerotic Dementia':ti,ab OR 'Arteriosclerotic Dementias':ti,ab OR 'Dementia, Arteriosclerotic':ti,ab OR 'Dementias, Arteriosclerotic':ti,ab |
| #11 | 'Acute Onset Vascular Dementia':ti,ab OR 'SubcORtical Vascular Dementia':ti,ab OR 'Dementia, SubcORtical Vascular':ti,ab OR 'Dementias, SubcORtical Vascular':ti,ab OR 'SubcORtical Vascular Dementias':ti,ab OR 'Vascular Dementia, SubcORtical':ti,ab OR 'Dementias, Vascular':ti,ab OR 'Vascular Dementias':ti,ab OR 'Vascular Dementia':ti,ab OR 'Vascular Dementia, Acute Onset':ti,ab OR 'Lacunar Dementia':ti,ab OR 'Dementia, Lacunar':ti,ab OR 'Dementias, Lacunar':ti,ab OR 'Lacunar Dementias':ti,ab |
| #12 | 'Dementias, Multiinfarct':ti,ab OR 'Multiinfarct Dementia':ti,ab OR 'Multiinfarct Dementias':ti,ab OR 'Multi-Infarct Dementia':ti,ab OR 'Multi Infarct Dementia':ti,ab OR 'Dementia Multi Infarct':ti,ab OR 'Dementia Multi-Infarcts':ti,ab OR 'Multi-Infarct,Dementia':ti,ab OR 'Multi-Infarcts, Dementia':ti,ab OR 'Dementia, Multiinfarct':ti,ab OR 'Dementia, Multi Infarct':ti,ab OR 'Dementias, Multi-Infarct':ti,ab OR 'Multi-Infarct Dementias':ti,ab OR 'Multiinfarct Dementia':ti,ab |
| #13 | 'Multiinfarct Dementia'/exp |
| #14 | 'Cognitive Dysfunction':ti,ab OR 'Cognitive Dysfunctions':ti,ab OR 'Dysfunction, Cognitive':ti,ab OR 'Dysfunctions, Cognitive':ti,ab OR 'Cognitive Impairments':ti,ab OR 'Cognitive Impairment':ti,ab OR 'Impairment, Cognitive':ti,ab OR 'Impairments, Cognitive':ti,ab OR 'Mild Cognitive Impairment':ti,ab OR 'Cognitive Impairment, Mild':ti,ab OR 'Cognitive Impairments, Mild':ti,ab OR 'Impairment, Mild Cognitive':ti,ab OR 'Impairments, Mild Cognitive':ti,ab OR 'Mild Cognitive Impairments':ti,ab OR 'Mild Neurocognitive DisORder':ti,ab OR 'DisORder, Mild Neurocognitive':ti,ab OR 'DisORders, Mild Neurocognitive':ti,ab OR 'Mild Neurocognitive DisORders':ti,ab OR 'Neurocognitive DisORder, Mild':ti,ab OR 'Neurocognitive DisORders, Mild':ti,ab OR 'Cognitive Decline':ti,ab OR 'Cognitive Declines':ti,ab OR 'Decline, Cognitive':ti,ab OR 'Declines, Cognitive':ti,ab OR 'Mental DeteriORation':ti,ab OR 'DeteriORation, Mental':ti,ab OR 'DeteriORations, Mental':ti,ab OR 'Mental DeteriORations':ti,ab |
| #15 | 'PSCI':ti,ab OR 'PSD':ti,ab OR 'PSCIND':ti,ab OR 'post stroke cognitive impairment':ti,ab OR 'cognitive impairment after stroke':ti,ab OR 'post stroke cognitive impairment no dementia':ti,ab OR 'post stroke dementia':ti,ab |
| #16 | #6 OR #7 OR #8 OR #9 OR #10 OR #11 OR #12 OR #13 OR #14 OR #15 |
| #17 | '3-n-butylphthalide'/exp |
| #18 | 'N-butylphthalide':ti,ab OR 'NBP':ti,ab OR 'Butylphthalide':ti,ab OR '(S)-(-)-3-butylphthalide':ti,ab OR '3-n-butylphthalide':ti,ab |
| #19 | #17 OR #18 |
| #20 | #5 AND #16 AND #19 |
| **The search strategy for CNKI** | |
| (SU = '中风' OR SU = '卒中' OR SU = '脑卒中' OR SU = '脑梗死' OR SU = '脑梗塞' OR SU = '脑栓塞' OR SU = '脑出血' OR SU = '蛛网膜下腔出血') AND (SU = '认知功能' OR SU = '认知障碍' OR SU = '认知受损' OR SU = '认知损伤' OR SU = '痴呆') AND (SU = '丁苯酞' OR SU = '恩必普' OR SU = '丁基苯酞') AND (FT= '随机') | |
| **The search strategy for Wanfang** | |
| 主题:((“丁苯酞” OR “恩必普” OR “丁基苯酞”) AND (“中风” OR “卒中” OR “脑卒中” OR “脑梗死” OR “脑梗塞” OR “脑栓塞” OR “脑出血” OR “蛛网膜下腔出血”) AND (“认知功能” OR “认知障碍” OR “认知受损” OR “认知损伤” OR “痴呆”) AND “随机”) | |
| **The search strategy for VIP** | |
| ((M=丁苯酞 OR 恩必普 OR 丁基苯酞) OR (R=丁苯酞 OR 恩必普 OR 丁基苯酞)) AND ((M=中风 OR 卒中 OR 脑卒中 OR 脑梗死 OR 脑梗塞 OR 脑栓塞 OR 脑出血 OR 蛛网膜下腔出血) OR (R=中风 OR 卒中 OR 脑卒中 OR 脑梗死 OR 脑梗塞 OR 脑栓塞 OR 脑出血 OR 蛛网膜下腔出血)) AND ((M=认知功能 OR 认知障碍 OR 认知受损 OR 认知损伤 OR 痴呆) OR (R=认知功能 OR 认知障碍 OR 认知受损 OR 认知损伤 OR 痴呆)) AND ((M=随机) OR (R=随机)) | |
| **The search strategy for SinoMed** | |
| (“丁苯酞”[常用字段:智能] OR “恩必普”[常用字段:智能] OR “丁基苯酞”[常用字段:智能]) AND ("中风"[常用字段:智能] OR "卒中"[常用字段:智能] OR "脑卒中"[常用字段:智能] OR "脑梗死"[常用字段:智能] OR "脑梗塞"[常用字段:智能] OR "脑栓塞"[常用字段:智能] OR "脑出血"[常用字段:智能] OR "蛛网膜下腔出血"[常用字段:智能]) AND ("认知功能"[常用字段:智能] OR "认知障碍"[常用字段:智能] OR "认知受损"[常用字段:智能] OR "认知损伤"[常用字段:智能] OR "痴呆"[常用字段:智能]) AND (随机对照试验[文献类型]) AND (人类[特征词]) | |

**1.2 Table S2 The list of excluded reports**

| **Reports excluded reason** | **Number** |
| --- | --- |
| Not meet intervention criteria | 2(Zhao, 2020; Zhao et al., 2018) |
| Duplicate or unclear data | 2(Mo, 2020; Xing and Abudushalamu, 2015) |
| Not meet outcome criteria | 3(Ma, 2020; Guo et al., 2020; Lin, 2019) |

**References**

Zhao, D. (2020). Effect of Butylphthalide combined with Citicoline in the treatment of cognitive impairment after stroke. Journal of North Pharmacy. 17(3), 140-178. doi: 10.3969/j.issn.1672-8351.2020.03.104

Zhao, S., Song, Z., Zhu, Y., Chen, Q., and Zhao, J. (2018). Effect of Butylphthalide combined with Piracetam in the treatment of cerebral infarction complicated with vascular cognitive impairment. China Health Care & Nutrition. 26, 167-168. doi: 10.3969/j.issn.1004-7484.2018.26.234

Mo, Z. (2020) Effect of Piracetam combined with Butylphthalide on cognitive impairment after stroke and its effect on Hcy. Heilongjiang Medicine Journal. 33(6), 1344-1345. doi: 10.14035/j.cnki.hljyy.2020.06.053

Xing, G., and Abudushalamu, A. (2015). Effect comparison of Butylphthalide and Nimodipine in the treatment of vascular dementia after stroke. Chin J Publ Heal. 31, 231+234.

Ma, J. (2020). Effect of Butylphthalide on vascular dementia after stroke. Diet Health. 43, 93.

Guo, Y., Li, D., and Xiao, C. (2020). Clinical value of butylphthalide capsule combined with piracetam in vascular dementia. Chin Comm Doc. 36(4), 56-57. doi: 10.3969/j.issn.1007-614x.2020.04.031

Lin, B. (2019). Effects of Butylphthalide combined with Oxiracetam on neurological and cognitive function of patients with cognitive impairment after cerebral infarction. Chin Comm Doc. 35(3), 50+52. doi: 10.3969/j.issn.1007-614x.2019.03.032

**1.3 Table S3 PRISMA 2020 checklist**

| **Section and Topic** | **Item #** | **Checklist item** | **Locationwhere item is reported** |
| --- | --- | --- | --- |
| **TITLE** | | |  |
| Title | 1 | Identify the report as a systematic review. | 1 |
| **ABSTRACT** | | |  |
| Abstract | 2 | See the PRISMA 2020 for Abstracts checklist. | 1-2 |
| **INTRODUCTION** | | |  |
| Rationale | 3 | Describe the rationale for the review in the context of existing knowledge. | 2 |
| Objectives | 4 | Provide an explicit statement of the objective(s) or question(s) the review addresses. | 2-3 |
| **METHODS** | | |  |
| Eligibility criteria | 5 | Specify the inclusion and exclusion criteria for the review and how studies were grouped for the syntheses. | 3-4 |
| Information sources | 6 | Specify all databases, registers, websites, organisations, reference lists and other sources searched or consulted to identify studies. Specify the date when each source was last searched or consulted. | 3 |
| Search strategy | 7 | Present the full search strategies for all databases, registers and websites, including any filters and limits used. | Table S1 |
| Selection process | 8 | Specify the methods used to decide whether a study met the inclusion criteria of the review, including how many reviewers screened each record and each report retrieved, whether they worked independently, and if applicable, details of automation tools used in the process. | 4 |
| Data collection process | 9 | Specify the methods used to collect data from reports, including how many reviewers collected data from each report, whether they worked independently, any processes for obtaining or confirming data from study investigators, and if applicable, details of automation tools used in the process. | 4 |
| Data items | 10a | List and define all outcomes for which data were sought. Specify whether all results that were compatible with each outcome domain in each study were sought (e.g. for all measures, time points, analyses), and if not, the methods used to decide which results to collect. | 3 |
|  | 10b | List and define all other variables for which data were sought (e.g. participant and intervention characteristics, funding sources). Describe any assumptions made about any missing or unclear information. | 4 |
| Study risk of bias assessment | 11 | Specify the methods used to assess risk of bias in the included studies, including details of the tool(s) used, how many reviewers assessed each study and whether they worked independently, and if applicable, details of automation tools used in the process. | 4 |
| Effect measures | 12 | Specify for each outcome the effect measure(s) (e.g. risk ratio, mean difference) used in the synthesis or presentation of results. | 4 |
| Synthesis methods | 13a | Describe the processes used to decide which studies were eligible for each synthesis (e.g. tabulating the study intervention characteristics and comparing against the planned groups for each synthesis (item #5)). | 4 |
|  | 13b | Describe any methods required to prepare the data for presentation or synthesis, such as handling of missing summary statistics, or data conversions. | 4 |
|  | 13c | Describe any methods used to tabulate or visually display results of individual studies and syntheses. | 4 |
|  | 13d | Describe any methods used to synthesize results and provide a rationale for the choice(s). If meta-analysis was performed, describe the model(s), method(s) to identify the presence and extent of statistical heterogeneity, and software package(s) used. | 4 |
|  | 13e | Describe any methods used to explore possible causes of heterogeneity among study results (e.g. subgroup analysis, meta-regression). | 4 |
|  | 13f | Describe any sensitivity analyses conducted to assess robustness of the synthesized results. | 4 |
| Reporting bias assessment | 14 | Describe any methods used to assess risk of bias due to missing results in a synthesis (arising from reporting biases). | 4 |
| Certainty assessment | 15 | Describe any methods used to assess certainty (or confidence) in the body of evidence for an outcome. | 5 |

| **Section and Topic** | **Item #** | **Checklist item** | **Location where item is reported** |
| --- | --- | --- | --- |
| **RESULTS** | | |  |
| Study selection | 16a | Describe the results of the search and selection process, from the number of records identified in the search to the number of studies included in the review, ideally using a flow diagram. | 5+Figure 1 |
|  | 16b | Cite studies that might appear to meet the inclusion criteria, but which were excluded, and explain why they were excluded. | Table S2 |
| Study characteristics | 17 | Cite each included study and present its characteristics. | 5+Table 1 |
| Risk of bias in studies | 18 | Present assessments of risk of bias for each included study. | 5-6+Figure 2+Figure S1 |
| Results of individual studies | 19 | For all outcomes, present, for each study: (a) summary statistics for each group (where appropriate) and (b) an effect estimate and its precision (e.g. confidence/credible interval), ideally using structured tables or plots. | 6-8+Figure 3-11 |
| Results of syntheses | 20a | For each synthesis, briefly summarise the characteristics and risk of bias among contributing studies. | 6-8+Figure 3-11 |
|  | 20b | Present results of all statistical syntheses conducted. If meta-analysis was done, present for each the summary estimate and its precision (e.g. confidence/credible interval) and measures of statistical heterogeneity. If comparing groups, describe the direction of the effect. | 6-8+Figure 3-11 |
|  | 20c | Present results of all investigations of possible causes of heterogeneity among study results. | 6-8+Figure 3-11 |
|  | 20d | Present results of all sensitivity analyses conducted to assess the robustness of the synthesized results. | 6-8+Figure 3-11 |
| Reporting biases | 21 | Present assessments of risk of bias due to missing results (arising from reporting biases) for each synthesis assessed. | 8+Figure 12 |
| Certainty of evidence | 22 | Present assessments of certainty (or confidence) in the body of evidence for each outcome assessed. | 8+Table 2-3 |
| **DISCUSSION** | | |  |
| Discussion | 23a | Provide a general interpretation of the results in the context of other evidence. | 8 |
|  | 23b | Discuss any limitations of the evidence included in the review. | 9-10 |
|  | 23c | Discuss any limitations of the review processes used. | 9-10 |
|  | 23d | Discuss implications of the results for practice, policy, and future research. | 8-9 |
| **OTHER INFORMATION** | | |  |
| Registration and protocol | 24a | Provide registration information for the review, including register name and registration number, or state that the review was not registered. | 3 |
|  | 24b | Indicate where the review protocol can be accessed, or state that a protocol was not prepared. | 3 |
|  | 24c | Describe and explain any amendments to information provided at registration or in the protocol. | 10 |
| Support | 25 | Describe sources of financial or non-financial support for the review, and the role of the funders or sponsors in the review. | 10 |
| Competing interests | 26 | Declare any competing interests of review authors. | 10 |
| Availability of data, code and other materials | 27 | Report which of the following are publicly available and where they can be found: template data collection forms; data extracted from included studies; data used for all analyses; analytic code; any other materials used in the review. | 10 |

*From:* Page MJ, McKenzie JE, Bossuyt PM, Boutron I, Hoffmann TC, Mulrow CD, et al. The PRISMA 2020 statement: an updated guideline for reporting systematic reviews. BMJ 2021;372:n71. doi: 10.1136/bmj.n71For more information, visit: <http://www.prisma-statement.org/>

**2 Supplemental figures**

**2.1 Figure S1: Risk of bias summary**


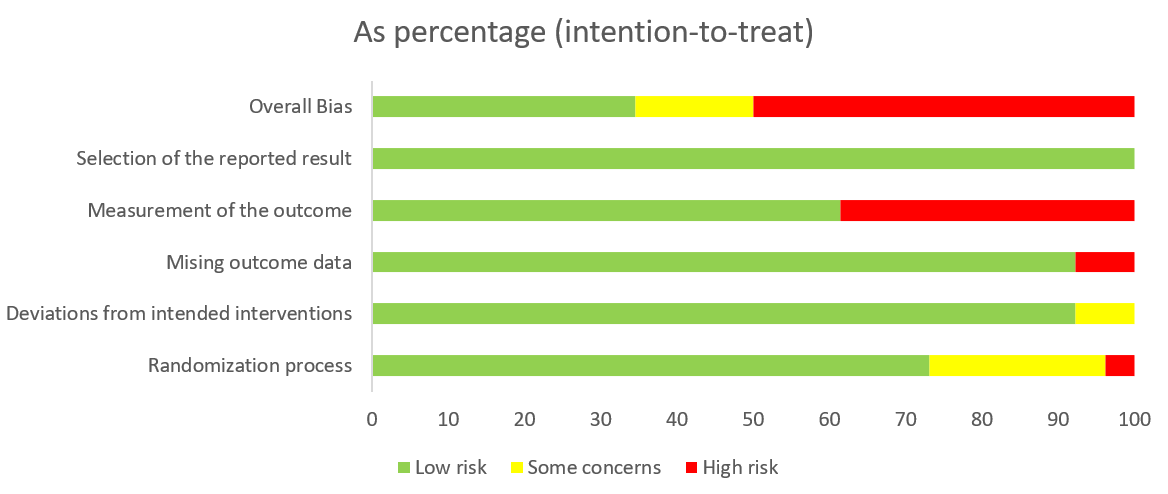

Supplement: Supplementary file 1 [file DataSheet1.docx]
